# Multisite Proton–Coupled Electron Transfer at a Keggin-Type Polyoxotungstate

**DOI:** 10.1021/jacs.5c18764

**Published:** 2026-02-03

**Authors:** Zhou Lu, Hania A. Guirguis, Ellen M. Matson

**Affiliations:** Department of Chemistry, 6927 University of Rochester, Rochester New York 14627, United States

## Abstract

Proton–coupled electron transfer (PCET) governs many redox transformations, but is thermodynamically constrained when proton and electron transfer occur at a single site. Here, we introduce a new multisite PCET (MS-PCET) platform, based on the Keggin-type polyoxotungstate, [VW_12_O_40_]^3–^ (**VW**_**12**_). Pairing **VW**_**12**_ with either Brønsted bases or acids yields reagent pairs with tunable effective bond dissociation free energies (BDFE_eff_) over 15 kcal mol^–1^, enabling both oxidative and reductive H atom transfer reactions. Kinetic studies on the oxidative pathway by using 2,4,6-^t^Bu_3_PhOH as a model hydrogen atom (H atom) donor reveal a product-like, entropy-dominated concerted proton–electron transfer (CPET) pathway from a preorganized hydrogen-bonded complex. By contrast, reductive H atom transfer reactions exhibit larger Δ*H* ^‡^ values, measurable kinetic isotope effects, and balanced Brønsted slope, consistent with synchronous CPET-type mechanism. Extension to N–H, O–H, and C–H substrates demonstrates the versatility of the **VW**_**12**_ MS-PCET platform for tunable (de)­hydrogenation.

## Graphical abstract

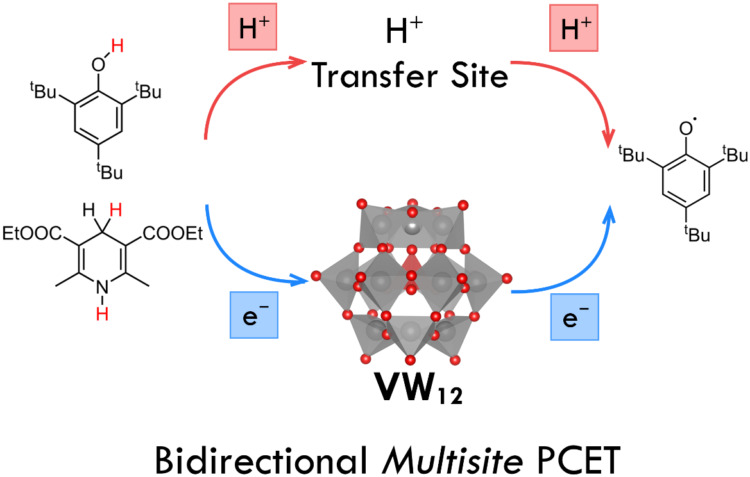

## Introduction

Proton–coupled electron transfer (PCET) is a fundamental chemical process underlying small molecule activation reactions central to energy conversion in both materials and biological systems (Figure 1).^1–6^ Within the PCET framework, the coupled transfer of an electron (e^–^) and a proton (H^+^) between donor and acceptor sites can proceed through a concerted proton–electron transfer (CPET) pathway, or by a sequence of stepwise processes (*e.g.*, electron transfer, ET; proton transfer, PT).^7,8^ The thermodynamic driving force for net hydrogen atom (H atom) transfer reactions is described by the difference in bond dissociation free energies (BDFE), which integrates the redox potential (*E*_1/2_) and acid dissociation constant (p*K*_a_), of the donor–acceptor pair. In canonical PCET systems, *E*_1/2_ and p*K*_a_ parameters are intrinsically correlated: modulation of one typically elicits a compensatory response of the other.^2,9^ This coupling constrains the independent tuning of BDFE values, limiting rational thermodynamic control over the reactivity and selectivity of H atom transfer processes.

**Figure 1:**
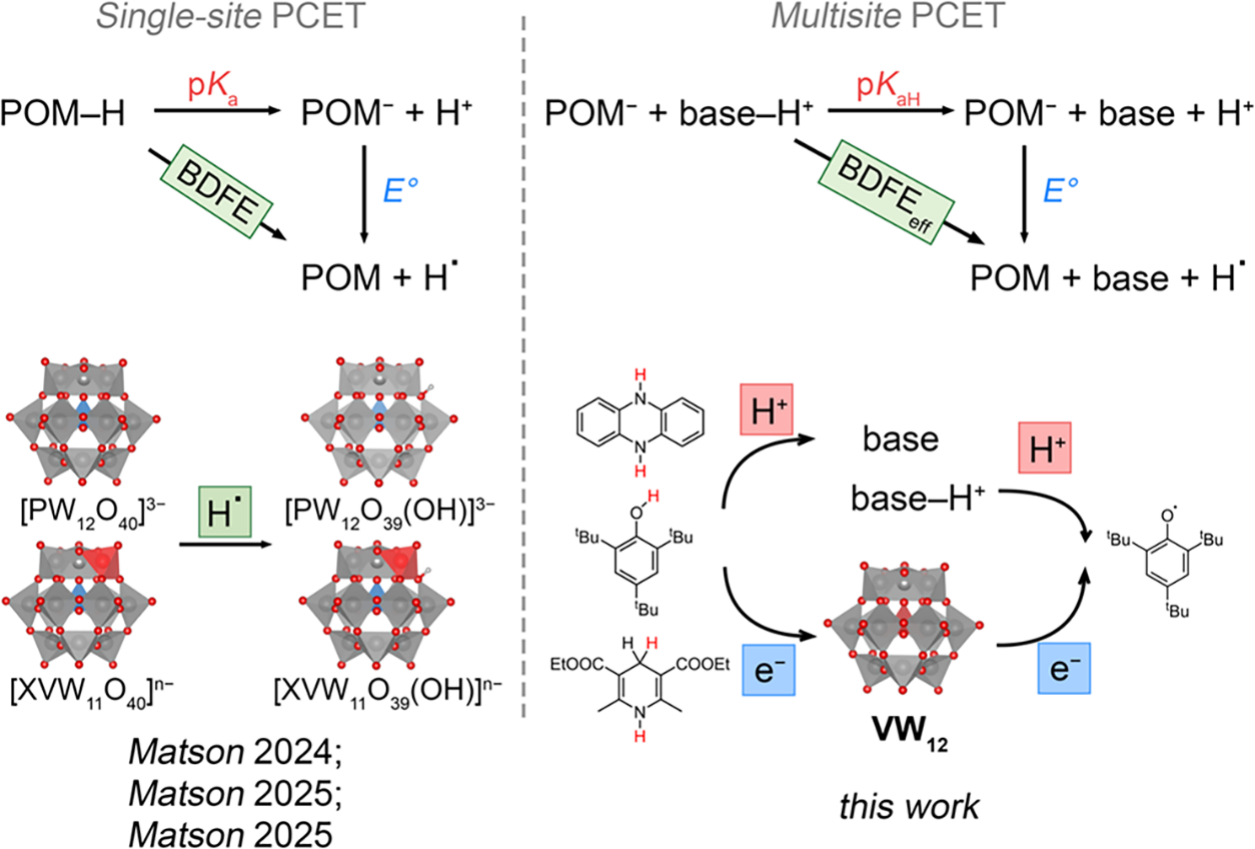
(Left) Summary of previously reported single site PCET process with polyoxometalates.^29–31^ X = Si, *n* = 5; X = P, *n* = 4; X = S, *n* = 3. (Right) Overview of multisite PCET process with Keggin-type [VW_12_O_40_]^3–^ studied in this work.

A straightforward conceptual strategy to overcome the intrinsic coupling of PT and ET energetics is to separate these processes at distinct molecular sites—a mechanistic design known as multisite PCET (MS-PCET; Figure 1).^2,10–15^ In this approach, discrete driving forces associated with PT and ET can be independently tuned through the selection of the proton donor/acceptor and the redox-active components. The thermodynamics of MS-PCET are described by an *effective* BDFE (BDFE_eff_), which is calculated using the p*K*_a_ of the donor and *E*_1/2_ of the redox center through the Bordwell Equation. Because ET and PT are spatially separated across two distinct reagents, the BDFE_eff_ can be tuned over a wide range of thermodynamic values than is accessible in conventional single-site PCET systems.

A growing number of experimental systems have leveraged MS-PCET to achieve controlled H atom transfer reactivity.^11,16–19^ The first clear example of MS-PCET was reported by Linschitz and co-workers; in this work, oxidation of phenols was achieved in the presence of pyridine (proton acceptor) and photogenerated C_60_ (*C_60_; electron acceptor).^20,21^ Building on this result, Knowles and co-workers combined an iridium photocatalytic mediator with a phosphate base to homolyze an amide N–H bond (BDFE­(N–H) = 99 kcal mol^–1^) en route to intramolecular hydroamidation of unactivated olefins.^22^ Mayer and co-workers demonstrated oxidative multisite PCET of 1-hydroxyl-2,2,6,6-tetramethylpiperidine (TEMPOH) by ferrocenium oxidants and pyridine bases, establishing a concerted PCET mechanism despite physical separation of proton and electron in the products.^10^ Most recently, Peters and co-workers reported a diaryl-*o*-carborane anion bearing an appended Brønsted acid to deliver H atom equivalents to fumarate, azoarene, and nitrotoluene; the authors note that competitive hydrogen evolution is observed owing to the extremely low BDFE_eff_ value of the system (31 kcal mol^–1^).^23^

Our research team has been investigating the thermodynamics and kinetics of PCET at polyoxometalate (POM) surfaces.^6,24–31^ POMs comprise a diverse class of redox-active metal-oxide assemblies that exhibit well-established proton-dependent electrochemical properties. These characteristics support conventional single-site PCET reactivity, with experimentally determined BDFE values between 40 and 70 kcal mol^–1^. In contrast, examples of MS-PCET in POM systems are rare. Results from Weinstock and co-workers demonstrated the formation of a protonated superoxide radical (HO_2_^•^) via ET from a one-electron-reduced polyoxotungstate (POT), [PW_12_O_40_]^4–^, followed by PT from a hydronium ion in water.^32^ In organic solvent, however, the surface of the one-electron-reduced assembly is sufficiently basic to interact with organic acids with low p*K*_a_ values, resulting in the formation of surface O–H bonds with low BDFE values (∼48 kcal mol^–1^).^29^ The low BDFE­(O–H) value translates to spontaneous evolution of H_2_, reducing the atom efficiency of hydrogenation chemistries possible within this single-site PCET system. Although interception of the hydrogen evolution pathway through the introduction of H atom acceptors has proven effective, complete suppression of hydrogen evolution is not possible.

With these limitations in mind, we hypothesized that a shift to a POT system with a redox-active moiety confined within a lattice of acidic tungsten oxide would result in an assembly surface insufficiently basic to interact with protons. Previously our research team has described a Keggin-type POT with redox-active vanadium center fully shielded by all-tungsten cage, [VW_12_O_40_]^3–^ (**VW**_**12**_), thus inhibiting the proton binding upon the reduction of the dopant.^30^ The constructed potential-p*K*_a_ (Pourbaix) diagram suggested the p*K*_a_ of [(H)­VW_12_O_40_]^2–^ is smaller than 5, sufficiently acidic to avoid interacting with protons in organic media. This design strategy would decouple electron and proton transfer, providing opportunities to interrogate MS-PCET reactivity.

Herein, we report a bidirectional MS-PCET platform based on a Keggin-type POT, **VW**_**12**_, in which electron and proton transfer are spatially decoupled and independently tunable. Pairing the appropriate redoxomer of **VW**_**12**_ with an external Brønsted base or acid results in MS-PCET reagents capable of either oxidative H atom abstraction or reductive H atom delivery. Using 2,4,6-^*t*^Bu_3_PhOH as a model H atom donor, systematic oxidative MS-PCET kinetics establish quantitative correlation between BDFE_eff_ and *k*_PCET_, revealing a concerted, product-like transition state from a strongly preorganized hydrogen-bonded assembly. Complementary kinetic analysis of the reductive hydrogenation of 2,4,6-^*t*^Bu_3_PhO^•^ uncovers a distinct mechanistic picture characterized by weaker preassociation, increased enthalpic contribution, a more synchronous CPET process. Extension of this platform to less polar C–H activation (Hantzsch ester) further demonstrates that MS-PCET reactivity is governed by the interplay of thermodynamic driving force, steric accessibility, and ground-state association. Collectively, these findings establish **VW**_**12**_ as a tunable and mechanistically informative MS-PCET scaffold capable of controlling H atom transfer reactivity and selectivity through the judicious selection of external acid/base partners.

## Results and Discussion

### Establishing Oxidative MS-PCET Reactivity for POT/Base Pairs

In previous work, we studied the reactivity between a lattice confined vanadium-dopant, **VW**_**12**_, and 5,10-dihydrophenazine (H_2_Phen).^30^ Isolation of the redox center with a cage of tungstate ions prevents interactions of protons with the more basic oxide ligands of the redox active vanadium center. This design element informs the reactivity of **VW**_**12**_ with H_2_Phen. Monitoring the reaction by electronic absorption spectroscopy reveals rapid oxidation of the substrate to its radical cationic form (H_2_Phen^+•^; λ = 446, 587, 646, and 709 nm) and reduction of the POT (**1e** ^**–**^ **-VW**_**12**_; λ = 740 nm) (Figure 2a–b). To expand upon this observation, we sought to establish a tunable platform that couples the isolated electron sponge **VW**_**12**_ with an *external* Brønsted base, thereby providing a proton acceptor site that would complete the PCET process without compromising the independence of the electron transfer. Within this MS-PCET mechanistic framework, traditional thermodynamics that describe H atom transfer are no longer suitable; instead, we calculate BDFE_eff_, including descriptors of reduction potentials and acid strength for the multicomponent system using the following equation

$${\mathrm{BDFE}}_{\mathrm{eff}}=23.06\times E_{1/2}\left(\mathrm{oxidant}\right)+1.37\times pK_{\mathrm{aH}}+C_{G}$$

where *E*_1/2_ is the redox potential of the electron transfer component (*E*_1/2_(**VW**_**12**_) = −0.10 V vs Fc^+/0^),^30^ p*K*_aH_ is the acidity of the conjugate acid of the added organic base,^33^ and C_G_ is a solvent-dependent constant (= 52.6 kcal mol^–1^ in acetonitrile, MeCN).^2^ This formalism allows quantification of the thermodynamics of H atom equivalent generated by the **VW**_**12**_/base reagent pair.

**Figure 2:**
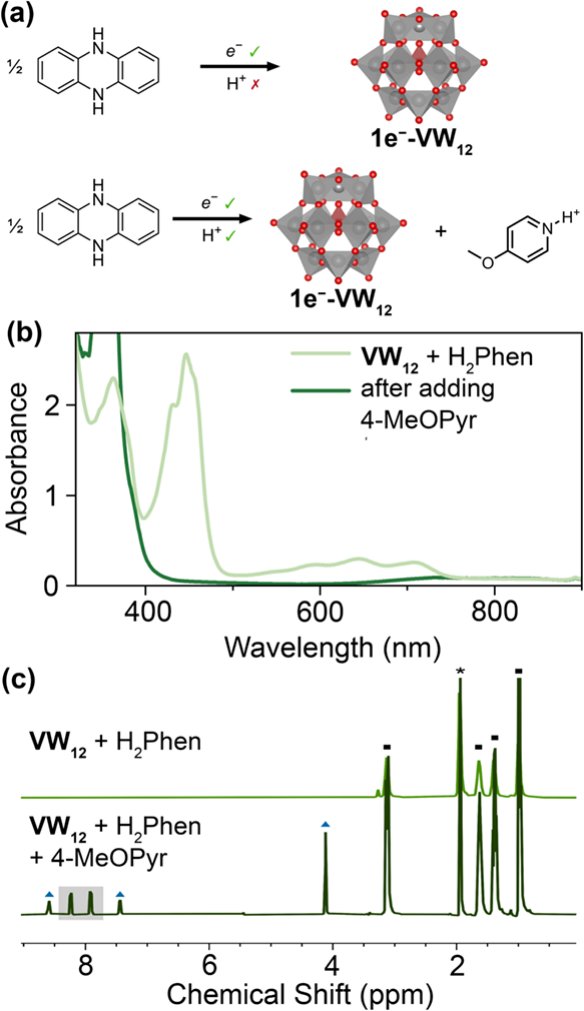
(a) Illustration of electron transfer from H_2_Phen to **VW**_**12**_ vs MS-PCET from H_2_Phen to **VW**_**12**_/4-MeOPyr pair. (b) Electronic absorption spectra and (c) stacked ^1^H NMR spectra of the reaction mixture in acetonitrile (MeCN) of **VW**_**12**_ and H_2_Phen before and after the addition of 4-MeOPyr in MeCN-*d*_3_, showing the disappearance of H_2_Phen^+•^ and the formation of Phen, highlighted in gray. The blue triangles and black squares represent the peaks of protonated 4-methoxypyridinium (4-MeOPyrH^+^) and tetrabutylammonium cations in **VW**_**12**_; the asterisk indicates the MeCN-*d*_3_.

Addition of an equivalent of 4-methoxypyridine (4-MeOPyr, p*K*_aH_ = 14.24 in MeCN)^33^ to the reaction mixture of **VW**_**12**_ and half equivalent of H_2_Phen results in the immediate loss of the intense green color of H_2_Phen^+•^ (Figure 2a−b). Analysis of the crude reaction mixture by ^1^H NMR spectroscopy reveals formation of the anticipated dehydrogenated product, phenazine (Phen), along with an equivalent of the protonated 4-methoxypyridinium cation (4-MeOPyrH^+^; Figure 2b). Reduction of the POT was confirmed through electronic absorption spectroscopy; a weak, broad band centered at 740 nm is observed, consistent with the formation of **1e^−^-VW**_**12**_.

The observed reactivity represents an oxidative MS-PCET reagent pair constructed by a Keggin-type POT **VW**_**12**_ (electron acceptor) and a separate Brønsted base, 4-MeOPyr (proton acceptor) with a BDFE_eff_ value of 70 kcal mol^–1^. Such decoupled pathways have been widely invoked in synthetic molecular and biological redox systems, including O–H/C–H bond activation, hydrogen evolution, and water oxidation.^12,19,34,35^ Notably, Mayer, Warren, and Hammes-Schiffer have established detailed thermodynamic and kinetic frameworks for such MS-PCET events in metal-oxo and metal-hydride systems, emphasizing the role of proton acceptor (p*K*_aH_) and electron acceptor (*E*_1/2_) in dictating reactivity.^5,36,37^ Within POM chemistry, however, MS-PCET remains largely unexplored, particularly in organic solvent. The above results expand this reactivity paradigm by demonstrating a distinct oxidative MS-PCET pair composed of **VW**_**12**_ and an organic base.

To experimentally probe the consequences of this modular MS-PCET design, we next investigated the oxidative dehydrogenation of 2,4,6-^*t*^Bu_3_PhOH (TTBP). Phenolic O–H bonds have long served as quantitative probes of PCET reactivity owing to their well-defined BDFE and established redox properties.^38,39^ The sterically encumbered TTBP variant, in particular, offers several advantages for delineating MS-PCET behavior; (1) its hindered structure (^*t*^Bu groups) suppresses competing bimolecular side reactions such as radical coupling, and (2) the relatively high BDFE­(O–H) (74.8 kcal mol^–1^ in MeCN)^2^ lies within the tunable range of the **VW**_**12**_/base pairs examined in this work.

To evaluate the oxidative capacity of the MS-PCET pair, we first examined the reaction between **VW**_**12**_ and a strong Brønsted base, 1,1,3,3-tetramethylguanidine (TMG; p*K*_aH_ = 23.35), toward TTBP.^33^ Given the fixed redox potential of **VW**_**12**_ in MeCN, this combination corresponds to a BDFE_eff_ of ≈82 kcal mol^–1^. Upon mixing of **VW**_**12**_, TMG, and TTBP in MeCN, an immediate and intense blue color is observed, indicative of *in situ* formation of the phenoxyl radical (Figure 3). Electronic absorption spectroscopy revealed diagnostic bands at 383, 400, and 626 nm, consistent with the reported spectrum of 2,4,6-^*t*^Bu_3_PhO^•^.^40^ An additional weak transition is observed at 740 nm, attributed to **1e** ^**–**^ **-VW**_**12**_. The disappearance of the phenolic O–H resonance in the ^1^H NMR spectrum and the appearance of a sharp isotropic EPR signal at *g* = 2.0023 (Figure 3b−c) further confirm the generation of the phenoxyl radical and reduced **1e** ^**–**^ **-VW**_**12**_.

**Figure 3:**
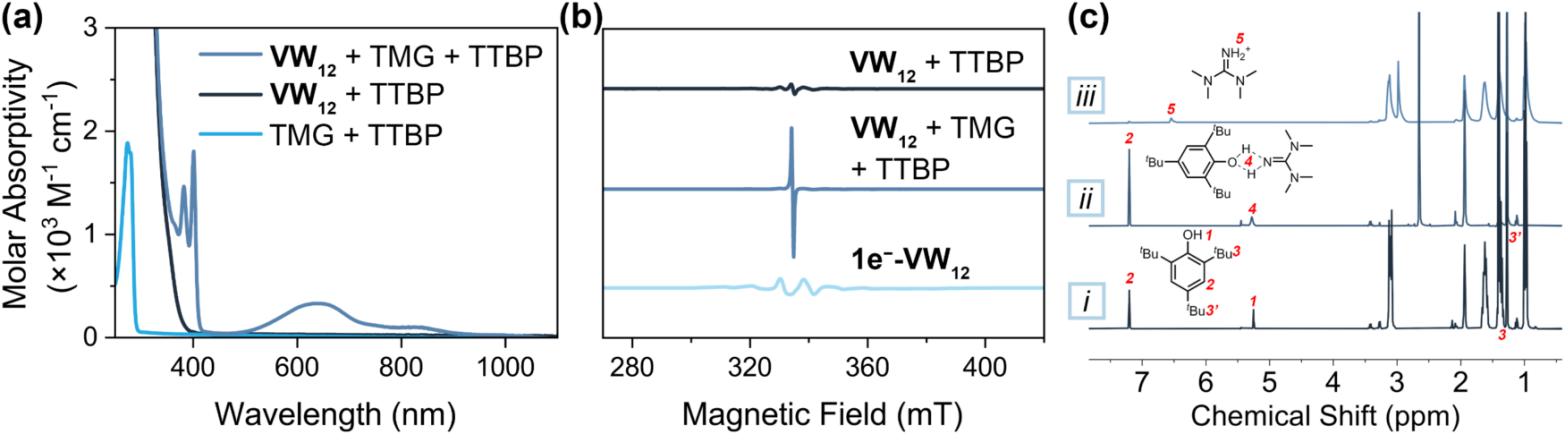
(a) Electronic absorption spectra and (b) X-band electron paramagnetic resonance (EPR) spectra of the equimolar mixture of TTBP and **VW**_**12**_/TMG reagent pair. (c) ^1^H NMR spectra of the equimolar reaction mixture of (i) TTBP and **VW**_**12**_, (ii) TTBP and TMG, and (iii) TTBP and **VW**_**12**_/TMG reagent pair in MeCN-*d*_3_.

Control experiments omitting either **VW**_**12**_ or TMG result in no observable dehydrogenation of substrate, establishing that both the POT and Brønsted base are essential for the overall transformation (Figure 3). ^51^V NMR spectrum of an equimolar **VW**_**12**_ and TMG mixture does not show additional signals and/or broadening, indicating that **VW**_**12**_ is stable in the presence of the organic base (Figure S1). However, the ^1^H NMR spectrum of the TTBP–TMG mixture exhibits a broadened resonance at δ = 5.3 ppm, integrating to two protons relative to the aromatic C–H signals (Figures 3c and S2). This new resonance forms concomitant with the disappearance of the terminal N–H resonance of TMG (Figures 3c and S2). This observation is consistent with the formation of a preassociated H-bonded pair that precedes electron transfer, facilitating concerted proton–electron transfer through a well-aligned donor–acceptor geometry.^41^

### Mechanistic Analysis of Oxidative MS-PCET Reactivity

To gain mechanistic insight into the oxidative MS-PCET process of H atom abstraction from TTBP, we attempted to monitor growth of the phenoxyl radical absorption at 400 nm to quantify reaction kinetics. However, the reaction between TTBP and **VW**_**12**_/TMG pair is found to be too rapid for analysis, proceeding to completion within 0.1 s at low temperature (Figure S3). According to the Bell–Evans–Polanyi (BEP) relationship, the rate constant of a reaction scales linearly with the overall free energy change (Δ*G*°) via modulation of the activation barrier (Δ*G* ^‡^).^42^ Consequently, to attenuate the reaction rate and access measurable kinetics, we employed weaker Brønsted bases to reduce the driving force of MS-PCET.

Thermochemical considerations based on the BDFE_eff_ of the **VW**_**12**_/base pairs guided the selection of bases with p*K*_aH_ values greater than 17, including primary, secondary, tertiary, and cyclic amines (Figures 4, S6–S13 and Table S1). The selected bases include *n*-butylamine (^*n*^BuNH_2_, p*K*_aH_ = 18.26), cyclohexylamine (CHA, p*K*_aH_ = 18.36), diethylamine (Et_2_NH, p*K*_aH_ = 18.75), diisopropylamine (^*i*^Pr_2_NH, p*K*_aH_ = 18.82), triethylamine (Et_3_N, p*K*_aH_ = 18.83), piperidine (p*K*_aH_ = 19.35), and pyrrolidine (p*K*_aH_ = 19.62).^33^ Upon addition of **VW**_**12**_ to equimolar solutions of TTBP and the respective base in MeCN, distinct color changes characteristic of 2,4,6-^*t*^Bu_3_PhO^•^ were observed, together with the appearance of **1e** ^**–**^ **-VW**_**12**_ and the conjugate acid of the relevant base.

**Figure 4:**
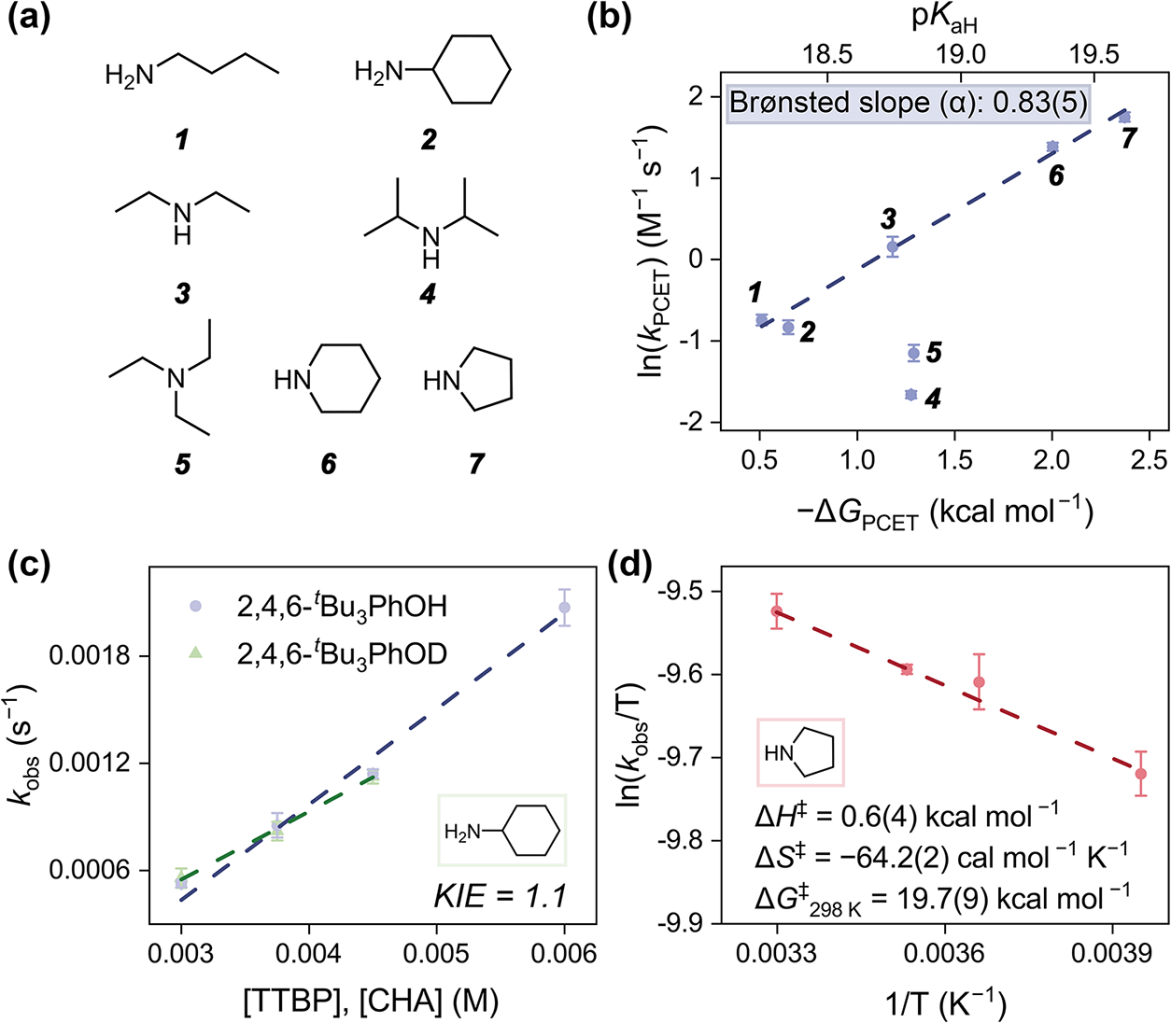
(a) The structures of all the Brønsted bases used in the oxidative MS-PCET reaction toward TTBP with **VW**_**12**_. (b) Plots of the second-order MS-PCET rate constant (*k*_PCET_) with respect to the p*K*_a_ values of the conjugated acids and MS-PCET reaction free energies (−Δ*G*_PCET_), where Δ*G*_PCET_ is obtained by subtracting BDFE­(O–H, TTBP) and BDFE_eff_(**VW**_**12**_/base). The data points with ^*i*^Pr_2_NH (***4*** in the Figure) and Et_3_N (***5***) are excluded from the linear fitting due to steric hindrance. The apparent slope is derived to be 1.40(8) and then translated to the Brønsted slope (α) of 0.83(5). (c) Plots of *k*_obs_ with respect to the concentrations of [2,4,6-^*t*^Bu_3_PhOH] or [2,4,6-^*t*^Bu_3_PhOD] with [CHA] at 20 °C, showing the KIE value of 1.1. (d) Eyring plot of the MS-PCET dehydrogenation reaction of TTBP in MeCN by 0.25 mM **VW**_**12**_/4.5 mM pyrrolidine between −20 and 30 °C.

As shown in Figure S4a, monitoring the absorbance growth of the phenoxyl radical at 400 nm results in single-exponential profiles, and fitting these traces affords the observed pseudo-first-order rate constants (*k*_obs_). Under conditions where [TTBP] and [base] are maintained at least 10-fold greater than [**VW**_**12**_], *k*_obs_ remains independent of [**VW**_**12**_] (Figure S4b), suggesting that the oxidant is not involved in the rate-determining step. In contrast, *k*_obs_ increases linearly with both the concentrations of base and TTBP (Figures S4c–d), demonstrating first-order dependence on each component. These results establish that the oxidative MS-PCET process proceeds through a bimolecular event involving the H atom donor and the Brønsted base (proton acceptor), while **VW**_**12**_ acts as a stoichiometric oxidant present in fixed concentration. As such, the slopes of the *k*_obs_ versus [TTBP] or [base] plots yield the overall second-order rate constants (*k*_PCET_, M^–1^ s^–1^), representing the intrinsic kinetics of the oxidative MS-PCET between **VW**_**12**_ and the phenol in the presence of a given base.

The oxidative dehydrogenation of TTBP by **VW**_**12**_ in the presence of various Brønsted bases proceeds at markedly different rates, which generally increase with the basicity of the proton acceptor. To rationalize this trend, the natural logarithm of the second-order rate constant (ln­(*k*_PCET_)) was plotted against both the p*K*_aH_ of the conjugate acids and the corresponding reaction free energies (−Δ*G*_PCET_) (Figure 4). The resulting linear correlation generally exhibits good agreement, suggesting that the driving force for PT plays a dominant role in governing the MS-PCET kinetics. However, two data points, corresponding to the rates of reaction in the presence of Et_3_N and ^*i*^Pr_2_NH, deviate substantially from the fit, exhibiting *k*_PCET_ values lower than those predicted from their Δ*G*_PCET_ values. We note that both bases possess bulky, alkyl substituents, which prevents the formation of a tightly bound donor/acceptor pair. This trait inhibits the overall rate of MS-PCET. Such steric modulation of PT kinetics has been observed previously in molecular MS-PCET systems.^43,44^

In considering the mechanism of MS-PCET, it is important to note that the p*K*_a_ of TTBP in MeCN (≈28) renders the corresponding stepwise proton transfer reaction endergonic (Δ*G*_PT_ > +9 kcal mol^–1^). This thermodynamic barrier rules out stepwise or proton-transfer-initiated mechanisms. Kinetic experiments with deuterated 2,4,6-^*t*^Bu_3_PhOD reveal a kinetic isotope effect (KIE) of 1.1 (Figures 4c and S18). This insignificant KIE value further indicates the PT step proceeds via the pre-equilibrated hydrogen-bonded complex and rules out the PT is a rate-limiting process. Despite this fact, a strong correlation between ln­(*k*_PCET_) and p*K*_aH_ is observed. Linear regression of ln­(*k*_PCET_) versus Δ*G*_PCET_ yields a slope (∂ln­(*k*_PCET_)/∂Δ*G*_PCET_) of −1.40(8), corresponding to a Brønsted slope (α) equal to 0.83(5). This value is greater than that expected for a symmetric transition state (α = 0.5), implicating a late, product-like transition state. It is worth noting that previously reported MS-PCET systems exhibit smaller α values (close to 0.5). For example, Mayer and co-workers reported α = 0.46 for the oxidation of TEMPOH by pyridine bases and ferrocenium oxidants, where proton and electron transfer contribute nearly equally to the rate constant.^10^ Bourrez, Hammarström, and co-workers observed a slope of (∂ ln­(*k*_PCET_)/∂Δ*G*°_PCET_) = 68 (meV)^−1^, corresponding to α ≈ 0.66, for a tungsten–hydride complex undergoing oxidative MS-PCET.^14^ Similarly, Mayer and Meyer have reported α ≈ 0.54 – 0.60 for the oxidation of pyridyl-substituted phenols and tyrosine residues, respectively.^45,46^ These α values close to 0.5 are indicative of the reactions transition states with synchronous proton and electron movement typical of concerted PCET processes. In contrast, the considerably higher α value (∼0.8) observed here suggests that the transition state for the **VW**_**12**_-mediated oxidation of TTBP is strongly product-like, and is possibly biased toward the proton-transfer component.

Given that the Brønsted slope of α = 0.83(5) is indicative of a late, product-like transition state, we sought to further probe the structure of the transition state of the rate-determining step of MS-PCET through variable-temperature kinetic analysis. To simplify the comparison, the most basic pyrrolidine with the fastest reaction rate was selected to derive activation parameters from Eyring plots (Figures 4d and S16). The Eyring analysis reveals the activation enthalpy (Δ*H* ^‡^) to be 0.6(4) kcal mol^–1^. This near-zero value suggests that the energy required for the bond-cleavage step does not dominate the thermodynamics of the activation barrier. In contrast, the derived activation entropy (Δ*S* ^‡^) affords a large negative value of −64.2(2) cal mol^–1^ K^–1^ and points to a highly ordered transition state. This large and negative entropic value is consistent with the formation of a pre-equilibrium hydrogen-bonded complex between the phenolic proton donor (TTBP) and the Brønsted base acceptor (pyrrolidine), as was inferred from spectroscopic evidence (Figure 3c). These activation parameters are notably smaller than those reported for single-site PCET oxidation of hydrazobenzene by other Keggin-type POTs, such as [PVW_11_O_40_]^4–^ and [SiVW_11_O_40_]^5–^.^30,31^ Indeed, decoupling the electron and proton acceptors appears to minimize the enthalpy for bond cleavage, while increasing the entropic penalty of the transition state to accommodate the three system components into a reaction-ready geometry. As such, the single site pathway is enthalpically controlled, whereas the oxidative multisite pathway is entropically controlled, relying on the preassociation of the donor/acceptor pair.

Having established the activation parameters and isotope effect for the oxidative MS-PCET with unhindered pyrrolidine, we next turn to evaluate how the sterically bulky base (e.g., Et_3_N) affects the dehydrogenation reaction and related transition state structure. Remarkedly, the Eyring parameters with Et_3_N (Δ*H* ^‡^ = 1.8(5) kcal mol^–1^, Δ*S* ^‡^ = −66.2(2) cal mol^–1^ K^–1^) are almost identical to those with pyrrolidine, suggesting that steric hindrance primarily attenuates the reaction rate rather than altering fundamental reaction mechanism (Figures S5a and S17). Although a slightly higher Δ*H* ^‡^ with Et_3_N is noticed, it is attributed to the influence of bulkiness on the protonation site, which is also reflected by the increased KIE value of 1.2 (Figures S5b and S19). Taken together, these thermodynamic and kinetic findings reinforce the mechanistic picture of a concerted MS-PCET process proceeding through a well-organized hydrogen-bonded transition state.

### Expanding the Scope of MS-PCET Reactivity of VW_12_

Beyond the N–H and O–H bond cleavages described above, activation of the less polar C–H bond represents an additional challenge of significant mechanistic interest. The oxidative **VW**_**12**_/base reagent pair provides a versatile platform for such transformations. To explore this type of reactivity, Hantzsch ester (HEH_2_) was selected as a model substrate. HEH_2_ functions as a convenient and well-characterized source of H-equivalents, donating H atoms from defined C–H and N–H sites with a BDFE near 70 kcal mol^–1^.^18^ This value lies within the tunable BDFE_eff_ window accessible to **VW**_**12**_/base combinations. Prior studies have shown that HEH_2_ reactivity is highly sensitive to the identity of the proton acceptor and the redox mediator, and ground state association effects, meaning that whether HEH_2_ engages in hydride or H atom transfer depends critically on mediator redox potential and on preorganization of the donor–acceptor pair.^18,47–49^ As such, HEH_2_ serves as a practical and informative benchmark for assessing whether **VW**_**12**_/base platform can achieve C–H activation through MS-PCET.

**VW**_**12**_, TMG, and half equiv of HEH_2_ were stirred in MeCN at room temperature overnight prior to solvent removal and diethyl ether extraction. The ^1^H NMR spectrum of the ether extract revealed the complete consumption of HEH_2_ and formation of Hantzsch pyridine (HE), including the diagnostic aromatic C–H signal at δ = 8.58 ppm (Figure 5a).^49^ This result establishes that the oxidative **VW**_**12**_/base MS-PCET reagent pair effects quantitative C–H dehydrogenation of HEH_2_ and expands the reactivity beyond conventional O–H and N–H substrates studied above.

**Figure 5:**
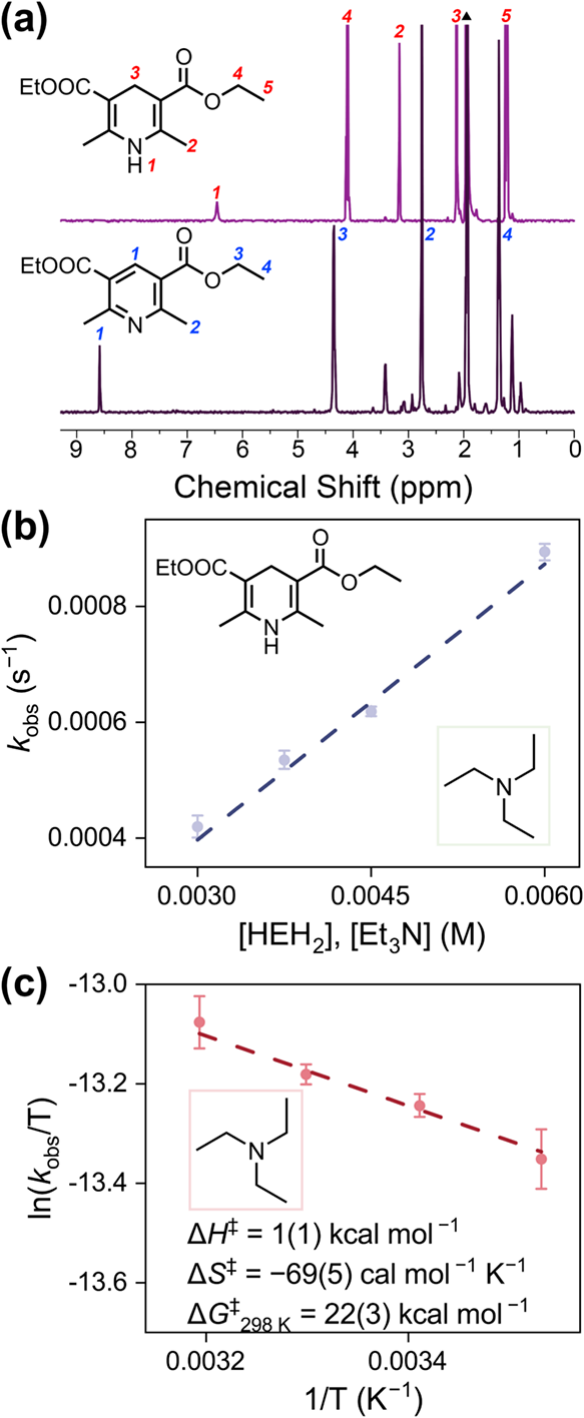
(a) ^1^H NMR spectra of (upper) Hantzsch ester (HEH_2_) and (bottom) the reaction mixture of HEH_2_ and **VW**_**12**_/TMG, showing the formation of Hantzsch pyridine (HE). The black triangle in the figure represents the solvent MeCN. (b) Plot of *k*_obs_ as a function of 3–6 mM [HEH_2_] and [Et_3_N]. (c) Eyring plots of the MS-PCET dehydrogenation reaction of HEH_2_ in MeCN by 0.25 mM **VW**_**12**_/3.75 mM Et_3_N between 0 and 30 °C.

To probe the mechanism, we followed product formation spectroscopically and extracted the kinetic parameters. Monitoring the absorption feature at 465 nm, concomitant appearance of HE (Figure S20), gives pseudo-first-order rate constant *k*_obs_ that varies linearly with the both concentrations of [HEH_2_] and [Et_3_N], yielding a second-order *k*_PCET_ value of 0.16(1) M^–1^ s^–1^ (Figures 5b and S21). Although the driving force Δ*G*_PCET_ is >6 kcal mol^–1^, the HEH_2_ oxidation proceeds more slowly than the corresponding TTBP dehydrogenation reactions with similar driving forces. We attribute the slower kinetics to weaker preassociation interactions between HEH_2_ and base. Indeed, HEH_2_ is a sterically congested, relatively poor hydrogen-bond donor at both the C–H or N–H sites, so alignment with the proton-accepting base is limited in comparison to the interactions between TTBP and base. Variable-temperature kinetic experiments return Eyring parameters with a near-zero activation enthalpy Δ*H* ^‡^ = 1(1) kcal mol^–1^ and a largely, negative activation entropy Δ*S* ^‡^ = −69(5) cal mol^–1^ K^–1^ (Figures 5c and S22), consistent with a concerted MS-PCET mechanism that requires an ordered, preorganized hydrogen-bonded assembly prior to H atom transfer.

Prototypical hydrocarbon H atom donors, like 9,10-dihydroanthracene and xanthene (BDFE­(C–H) = 72.9 and 70.2 kcal mol^–1^ in DMSO, respectively^2^) that lack polar E–H bonds show no reactivity under identical conditions (Figure S23). This outcome aligns with literature reports that conclude that the activation of nonpolar C–H bonds requires either large driving forces or a dedicated radical mediator, mechanisms distinct from the preorganized MS-PCET pathway invoked for the POM/base pair.^50–54^ In the absence of an N–H or other polar motif to facilitate preassociation of substrate and base, the **VW**_**12**_/base reagent pair lacks the geometric and energetic prerequisites to activate nonpolar C–H bonds.

### Bidirectional MS-PCET Reactivity with VW_12_: Investigations into Reductive MS-PCET

The direction of the observed MS-PCET reactivity can be inverted to construct a reductive reagent pair by combining the one-electron-reduced cluster, **1e** ^**–**^ **-VW**_**12**_, with an organic acid. **1e** ^–^ **-VW**_**12**_ was independently synthesized via reduction of **VW**_**12**_ with [^*n*^Bu_4_N]­BH_4_ in MeCN, affording a pale-green solid.^30^ The BDFE_eff_(**1e** ^**–**^ **-VW**_**12**_, 4-MeOPyrH^+^) is estimated to be 70 kcal mol^–1^, comparable to common reductive H atom donors.^2^ When 2,4,6-^*t*^Bu_3_PhO^•^ is treated with the **1e** ^**–**^ **-VW**_**12**_/4-MeOPyrH^+^ pair in MeCN, the characteristic blue color of the radical vanishes within 30 min, accompanied with the emergence of the phenolic O–H signal at 5.25 ppm in ^1^H NMR spectrum, indicating formation of TTBP (Figure 6a and S24).

**Figure 6:**
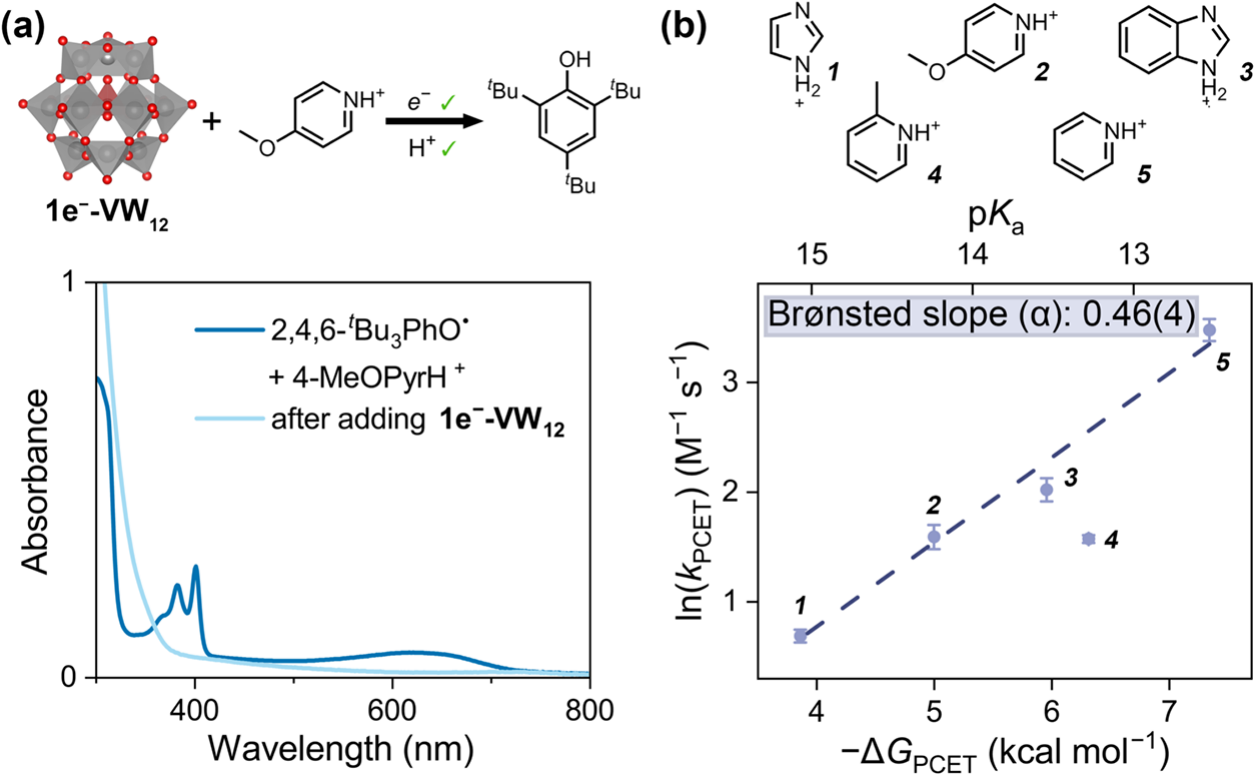
(a) Illustration of reductive MS-PCET from **1e** ^**–**^ **-VW**_**12**_/4-MeOPyrH^+^ pair to 2,4,6-^*t*^Bu_3_PhO^•^; electronic absorption spectra of the equimolar reaction mixture of 2,4,6-^*t*^Bu_3_PhO^•^ and 4-MeOPyrH^+^ before and after the addition of **1e** ^**–**^ **-VW**_**12**_, suggesting the reductive MS-PCET hydrogenation of 2,4,6-^*t*^Bu_3_PhO^•^ to afford TTBP. (b) The structures of all the Brønsted acids used in the reductive MS-PCET toward 2,4,6-^*t*^Bu_3_PhO^•^ with **1e** ^**–**^ **-VW**_**12**_; plots of the second-order MS-PCET rate constant (*k*_PCET_) with respect to acids’ p*K*_a_ and MS-PCET reaction free energies (−Δ*G*_PCET_), where Δ*G*_PCET_ is obtained by subtracting BDFE_eff_(**1e** ^**–**^ **-VW**_**12**_/acid) and BDFE­(O–H, TTBP). The data point with 2-PicH^+^ (***4*** in the Figure) is excluded from the linear fitting due to steric hindrance. The apparent slope is derived to be 0.77(6) and then translated to the Brønsted slope (α) of 0.46(4).

To probe the mechanism of reductive MS-PCET, we mirrored the kinetic studies performed for the oxidative process, monitoring the decay of the phenoxyl radical absorption at 626 nm. Linear dependence of *k*_obs_ on the concentrations of [2,4,6-^*t*^Bu_3_PhO^•^] or [acid] results in overall second-order PCET rate constants for the reaction *k*_PCET_ (M^–1^ s^–1^). A series of Brønsted acids, [imidazolium]­(BF_4_^–^) (ImH_2_^+^, p*K*_a_ = 15.07), 4-MeOPyrH^+^(BF_4_^–^) (p*K*_a_ = 14.24), [benzimidazolium]­(BF_4_^–^) (BimH_2_^+^, p*K*_a_ = 13.54), [2-picolinium]­(BF_4_^–^) (2-PicH^+^, p*K*_a_ = 13.28), and [pyridinium]­(BF_4_^–^) (PyrH^+^, p*K*_a_ = 12.56), were surveyed (Figure 6b and Table S1). As with the oxidative process, *k*_PCET_ generally increases with the acid strength; plotting ln­(*k*_PCET_) against p*K*_a_ values of organic acids and free energies (−Δ*G*_PCET_) gives good linearity (*R* ^2^ = 0.98, Figures 6b and S24–S29). The sole outlier is 2-PicH^+^, whose steric bulk adjacent to the protonation site produces a substantially slower rate than predicted, implicating steric inhibition of proton delivery.

The reductive series yields ∂ln­(*k*_PCET_)/∂Δ*G*_PCET_ = −0.77(6), corresponding to a Brønsted slope (α) of 0.46(4) (Figure 6b). This value, close to 0.5, suggests a balanced, transition state. The activation parameters for the reductive process derived from Eyring analysis with 4-MeOPyrH^+^ (Δ*H* ^‡^ = 6(2) kcal mol^–1^, Δ*S* ^‡^ = −50(5) cal mol^–1^ K^–1^), together with a moderate primary KIE of 1.6 (Figures S31–S33), are concordant with the following picture that the transition state retrains appreciable bond-cleavage character (enthalpic penalty) while requiring modest preorganization of the reacting pair.

By contrast, the oxidative process described above possesses a substantially larger Brønsted slope (α = 0.83(5)), a near-zero activation enthalpy (Δ*H* ^‡^ ≈ 0 kcal mol^–1^), a much more negative activation entropy (Δ*S* ^‡^ ≈ −65 cal mol^–1^ K^–1^), and a negligible KIE (∼1). These observables point to a product-like, transition state arising from a strongly preorganized hydrogen-bonded complex in which the ET is effectively stabilized by the preassociation; the overall rate is consequently highly sensitive to the driving force of PT. The dichotomy of kinetic descriptors for the two reaction directions reflects the differences in donor–acceptor interactions. Oxidative MS-PCET employs neutral, hydrogen-bonding bases that form tight, directional preassociations with TTBP, producing an entropy-controlled CPET. Reductive MS-PCET, however, adopts charged pyridinium or azolium acids that are both strongly solvated and prone to ion-pairing with the anionic **1e** ^**–**^ **-VW**_**12**_. Such solvation and ion-pairing effect attenuates direct hydrogen-bonding to the neutral phenoxyl radical, diminishing the importance of preassociation, and shifts a larger fraction of the barrier onto the PT coordinate.

Comparing multisite and single-site PCET clarifies why **VW**_**12**_ platform occupies a distinct mechanistic regime. In single-site PCET, commonly encountered at metal-oxo centers or on the surface site of POMs, redox and protonation properties are intrinsically coupled such that tuning one parameter tends to compensate the other.^6^ As a result, the accessible BDFE window is narrow, and activation barriers are often dominated by enthalpic reorganization at the redox locus. Multisite PCET naturally decouples those descriptors, allowing BDFE_eff_ to span over a wider range and permitting the reaction coordinate to move between enthalpy- and entropy-controlled regimes. Our results with **VW**_**12**_ exemplify this divergence; oxidative reactions proceed from tightly preassociated, entropy-dominated complexes that give product-like transition states, whereas the reductive direction displays larger enthalpic penalties and weaker preassociation consistent with a bond-cleavage sensitive transition state. This behavior contrasts with prior single-site POM studies in which substantial enthalpic barriers and competing protonation of oxide ligands frequently lead to hydrogen evolution or rigid driving-force and rate correlations.^24,26,29–31,55,56^ In this light, the **VW**_**12**_ platform extends POM reactivity by providing control over thermodynamics and molecule-level pathways.

Together, these oxidative and reductive transformations demonstrate the bidirectionality of MS-PCET with **VW**_**12**_, simply through toggling the redox state of the cluster and the acidity of the paired base to reversibly switch either H atom abstraction or delivery. This switch not only inverts the direction of net H atom transfer but also reshapes the activation energy landscape, altering the relative contributions of enthalpy and entropy and changing the extent of proton-transfer character at the transition state. Thus, by selecting acid/base identity, tuning acidity and steric profile, and controlling solvation or ion-pairing interactions, one can independently modulate both thermodynamic driving force (BDFE_eff_) and operative molecular pathway.

## Conclusion

In summary, this work establishes a modular Keggin-type polyoxotungstate–based platform that enables both oxidative and reductive *multisite* proton–coupled electron transfer (MS-PCET) by coupling the redox-active [VW_12_O_40_]^3–^ (**VW**_**12**_) cluster with external Brønsted bases or acids. This design decouples the electron and proton donors/acceptors, allowing the overall thermodynamic driving force to be tuned through the basicity/acidity of the proton acceptor/donor while maintaining a fixed redox potential of the POT. Systematic kinetic analyses in both directions of net H atom transfer reveal clear linear free-energy relationships between *k*_PCET_ and Δ*G*_PCET_, but distinct character of the transition-states of oxidative and reductive pathways. The kinetics of the oxidative pathway are dictated by entropy, exhibiting a largely negative Δ*S* ^‡^ (≈ −65 cal mol^–1^ K^–1^), a near-zero Δ*H* ^‡^, a Brønsted slope α = 0.83, and negligible KIE; collectively, these reaction descriptors are consistent with a tightly preassociated, product-like transition state of CPET, resulting from the formation of strong H-bonding interactions between substrate and base. Reductive MS-PCET exhibits a less negative Δ*S* ^‡^ (≈ −50 cal mol^–1^ K^–1^), larger Δ*H* ^‡^ (≈ 6 kcal mol^–1^), α = 0.46, and a measurable KIE of 1.6, indicators consistent with synchronous CPET that is limited by PT and weaker precursor association. Taken together, these results push POM chemistry beyond single-site reactivity and provide a molecular framework for designing H atom transfer systems in which redox and acid–base components are tuned independently to control both thermodynamics and mechanisms.

## Supplementary Material



## Notes

Experimental details, details about kinetics data, and results of kinetic investigations (PDF)
