# Supplementary material for: Multisite Proton–Coupled Electron Transfer at a Keggin-Type Polyoxotungstate
Source: J Am Chem Soc. 2026 Feb 3;148(6):6280–8. doi: 10.1021/jacs.5c18764 (PMC12921855; doi:10.1021/jacs.5c18764)
Supplement: Supplementary file 1 [file ja5c18764_si_001.pdf]

Supporting Information for

Multisite Proton–Coupled Electron Transfer at a Keggin-Type Polyoxotungstate

Zhou Lu,* Hania A. Guirguis, and Ellen M. Matson*

Department of Chemistry, University of Rochester, Rochester NY 14627, USA.

E-mail: zhoulu@rochester.edu (Z.L.), matson@chem.rochester.edu (E.M.M.)

Table of Contents

General Considerations and Physical Measurements. 5

Kinetics analysis 5

Table S1. Brønsted bases used in this work.[6] 6

Figure S1. ^{51}V NMR spectrum of equimolar $\mathbf{VW_{12}}$ and TMG in MeCN-d_3, showing the stability of $\mathbf{VW_{12}}$ in the presence of a strong organic base. 7

Figure S2. ^{1}H NMR spectrum of equimolar TTBP and TMG in MeCN-d_3, showing the formation of hydrogen-bonded adduct 7

Figure S3. Plot of absorbance at 400 nm over time for the oxidative MS-PCET reaction of dehydrogenation of TTBP between 0.02 mM $\mathbf{VW_{12}}$ and 2 mM [TTBP]/[TMG] in MeCN at −30 °C 8

Figure S4. (a) Determination of the observed pseudo-first-order rate constant for the oxidative MS-PCET reaction of TTBP (3 mM) by $\mathbf{VW_{12}}$ (0.25 mM)/pyrrolidine (3 mM) in MeCN at 20 °C by monitoring the absorbance change at 400 nm over the time. Gray dots are raw absorbance data points and the red line is the single exponential fitted trace. (b) Plot of log(k_{obs}) with respect to log([$\mathbf{VW_{12}}$]). The concentrations of [TTBP] and [pyrrolidine] are held constant at 4.5 mM. (c) Plot of log(k_{obs}) with respect to log([CHA]). The concentrations of [$\mathbf{VW_{12}}$] and [TTBP] are held constant at 0.25 and 3 mM, respectively. (d) Plot of k_{obs} as the function of [TTBP] and [pyrrolidine]. The concentration of [$\mathbf{VW_{12}}$] is held constant at 0.25 mM. 9

Figure S5. (a) Eyring plots of the MS-PCET dehydrogenation reaction of TTBP in MeCN by 0.25 mM $\mathbf{VW_{12}}$/5 mM Et_3N between 10 and 40 °C. (b) Plots of k_{obs} with respect to the concentrations of [2,4,6-^{t}Bu_3PhOH] or [2,4,6-^{t}Bu_3PhOD] with [Et_3N] at 30 °C, showing the KIE value of 1.2. The concentration of [$\mathbf{VW_{12}}$] was held constant at 0.25 mM. 10

Figure S6. Plots of absorbance at 400 nm over time for the oxidative MS-PCET reaction of dehydrogenation of TTBP between 0.25 mM $\mathbf{VW_{12}}$ and varied concentrations of [TTBP]/[^{n}BuNH_2] in MeCN at 20 °C with (gray) raw data and (red) fitting curve, along with fit-derived k_{obs}. 11

Figure S7. Plots of absorbance at 400 nm over time for the oxidative MS-PCET reaction of dehydrogenation of TTBP between 0.25 mM $\mathbf{VW_{12}}$ and varied concentrations of [TTBP]/[CHA] in MeCN at 20 °C with (gray) raw data and (red) fitting curve, along with fit-derived k_{obs}. 12

Figure S8. Plots of absorbance at 400 nm over time for the oxidative MS-PCET reaction of dehydrogenation of TTBP between 0.25 mM $\mathbf{VW_{12}}$ and varied concentrations of [TTBP]/[Et_2NH] in MeCN at 20 °C with (gray) raw data and (red) fitting curve, along with fit-derived k_{obs}. 13

Figure S9. Plots of absorbance at 400 nm over time for the oxidative MS-PCET reaction of dehydrogenation of TTBP between 0.25 mM $\mathbf{VW_{12}}$ and varied concentrations of [TTBP]/[^{i}Pr_2NH] in MeCN at 20 °C with (gray) raw data and (red) fitting curve, along with fit-derived k_{obs}. 14

Figure S11. Plots of absorbance at 400 nm over time for the oxidative MS-PCET reaction of dehydrogenation of TTBP between 0.25 mM $\mathbf{VW_{12}}$ and varied concentrations of [TTBP]/[piperidine] in MeCN at 20 °C with (gray) raw data and (red) fitting curve, along with fit-derived k_{obs}. 16

Figure S12. Plots of absorbance at 400 nm over time for the oxidative MS-PCET reaction of dehydrogenation of TTBP between 0.25 mM $\mathbf{VW_{12}}$ and varied concentrations of [TTBP]/[pyrrolidine] in MeCN at 20 °C with (gray) raw data and (red) fitting curve, along with fit-derived k_{obs}. 17

Figure S13. Plots of k_{obs} versus the concentrations of TTBP and different bases. 18

Figure S14. Plots of absorbance at 400 nm over time for the oxidative MS-PCET reaction of dehydrogenation of TTBP between varied concentrations of $\mathbf{VW_{12}}$ and 4.5 mM [TTBP]/[pyrrolidine] in MeCN at 20 °C with (gray) raw data and (red) fitting curve, along with fit-derived k_{obs}. 19

Figure S15. Plots of absorbance at 400 nm over time for the oxidative MS-PCET reaction of dehydrogenation of TTBP between 0.25 mM $\mathbf{VW_{12}}$, 3 mM TTBP, and varied concentrations of [CHA] in MeCN at 20 °C with (gray) raw data and (red) fitting curve, along with fit-derived k_{obs} 20

Figure S16. Plots of absorbance at 400 nm over time for the oxidative MS-PCET reaction of dehydrogenation of TTBP between 0.25 mM $\mathbf{VW_{12}}$ and 4.5 mM [TTBP]/[pyrrolidine] in MeCN at varied temperature between −20 and 30 °C with (gray) raw data and (red) fitting curve, along with fit-derived k_{obs}. 21

Figure S17. Plots of absorbance at 400 nm over time for the oxidative MS-PCET reaction of dehydrogenation of TTBP between 0.25 mM $\mathbf{VW_{12}}$ and 5 mM [TTBP]/[Et_3N] in MeCN at varied temperature between 10 and 40 °C with (gray) raw data and (red) fitting curve, along with fit-derived k_{obs}. 22

Figure S18. Plots of absorbance at 400 nm over time for the oxidative MS-PCET reaction of dehydrogenation of 2,4,6-^{t}Bu_3PhOD between 0.25 mM $\mathbf{VW_{12}}$ and varied concentrations of [2,4,6-^{t}Bu_3PHOD]/[pyrrolidine] in MeCN at 20 °C with (gray) raw data and (red) fitting curve, along with fit-derived k_{obs}. 23

Figure S19. Plots of absorbance at 400 nm over time for the oxidative MS-PCET reaction of dehydrogenation of 2,4,6-^{t}Bu_3PhOD between 0.25 mM $\mathbf{VW_{12}}$ and varied concentrations of [2,4,6-^{t}Bu_3PhOD]/[Et_3N] in MeCN at 30 °C with (gray) raw data and (red) fitting curve, along with fit-derived k_{obs}. 24

Figure S20. Electronic absorption spectra of 0.5 equiv Hantzsch ester (HEH_2) and 1 equiv Et_3N before and after the addition of 1 equiv $\mathbf{VW_{12}}$ over the time from 0 to 45 minutes. 25

Figure S21. Plots of absorbance at 465 nm over time for the oxidative MS-PCET reaction of dehydrogenation of Hantzsch ester (HEH_2) between 0.25 mM $\mathbf{VW_{12}}$ and varied concentrations of [HEH_2]/[Et_3N] in MeCN at 20 °C with (gray) raw data and (red) fitting curve, along with fit-derived k_{obs}. 26

Figure S22. Plots of absorbance at 465 nm over time for the oxidative MS-PCET reaction of dehydrogenation of Hantzsch ester (HEH_2) between 0.25 mM $\mathbf{VW_{12}}$ and 3.75 mM [HEH_2]/[Et_3N] in MeCN at varied temperature between 0 and 30 °C with (gray) raw data and (red) fitting curve, along with fit-derived k_{obs}. 27

Figure S23. ^{1}H NMR of the reaction mixture of $\mathbf{VW_{12}}$/TMG reagent pair with (a) 9,10-dihydroanthracene and (b) xanthene in MeCN-d_3, while showing no reactivity. 28

Figure S24. ^{1}H NMR of the reaction mixture of (blue) 2,4,6-^{t}Bu_3PhO$^\bullet$ radical, (green) 2,4,6-^{t}Bu_3PhO$^\bullet$ radical and 4-MeOPyrH$^+$, and (red) 2,4,6-^{t}Bu_3PhO$^\bullet$ radical, 4-MeOPyrH$^+$, and $\mathbf{1e^–\text{-}VW_{12}}$ in MeCN-d_3, showing the formation of TTBP with O–H signal highlighted in blue. The asterisk indicates the trace impurity of dichloromethane. 29

Figure S25. Plots of absorbance at 626 nm over time for the reductive MS-PCET reaction of hydrogenation of 2,4,6-^{t}Bu_3PhO$^\bullet$ between 0.25 mM $\mathbf{VW_{12}}$ and varied concentrations of [2,4,6-^{t}Bu_3PhO$^\bullet$]/[4-MeOPyrH$^+$($BF_4^–$)] in MeCN at 20 °C with (gray) raw data and (red) fitting curve, along with fit-derived k_{obs}. 30

Figure S26. Plots of absorbance at 626 nm over time for the reductive MS-PCET reaction of hydrogenation of 2,4,6-^{t}Bu_3PhO$^\bullet$ between 0.25 mM $\mathbf{VW_{12}}$ and varied concentrations of [2,4,6-^{t}Bu_3PhO$^\bullet$]/[PyrH$^+$($BF_4^–$)] in MeCN at 20 °C with (gray) raw data and (red) fitting curve, along with fit-derived k_{obs}. 31

Figure S27. Plots of absorbance at 626 nm over time for the reductive MS-PCET reaction of hydrogenation of 2,4,6-^{t}Bu_3PhO$^\bullet$ between 0.25 mM $\mathbf{VW_{12}}$ and varied concentrations of [2,4,6-^{t}Bu_3PhO$^\bullet$]/[2-PicH$^+$($BF_4^–$)] in MeCN at 20 °C with (gray) raw data and (red) fitting curve, along with fit-derived k_{obs}. 32

Figure S28. Plots of absorbance at 626 nm over time for the reductive MS-PCET reaction of hydrogenation of 2,4,6-^{t}Bu_3PhO$^\bullet$ between 0.25 mM $\mathbf{VW_{12}}$ and varied concentrations of [2,4,6-^{t}Bu_3PhO$^\bullet$]/[BimH$_2^+$($BF_4^–$)] in MeCN at 20 °C with (gray) raw data and (red) fitting curve, along with fit-derived k_{obs}. 33

Figure S29. Plots of absorbance at 626 nm over time for the reductive MS-PCET reaction of hydrogenation of 2,4,6-^{t}Bu_3PhO$^\bullet$ between 0.25 mM $\mathbf{VW_{12}}$ and varied concentrations of [2,4,6-^{t}Bu_3PhO$^\bullet$]/[ImH$_2^+$($BF_4^–$)] in MeCN at 20 °C with (gray) raw data and (red) fitting curve, along with fit-derived k_{obs}. 34

Figure S30. Plots of k_{obs} versus the concentrations of 2,4,6-^{t}Bu_3PhO$^\bullet$ and different acids. 35

Figure S31. (a) Plots of k_{obs} with respect to the concentrations of [2,4,6-^{t}Bu_3PhO$^\bullet$] with [4-MeOPyrH$^+$($BF_4^–$)] or [4-MeOPyrD$^+$(OTf$^–$)] (OTf = trifluoromethanesulfonate) at 20 °C, showing the KIE value of 1.6. (b) Eyring plots of the MS-PCET hydrogenation reaction of 2,4,6-^{t}Bu_3PhO$^\bullet$ in MeCN by 0.25 mM $\mathbf{1e^–\text{-}VW_{12}}$/3 mM 4-MeOPyrH$^+$ between 0 and 20 °C. 36

Figure S32. Plots of absorbance at 626 nm over time for the reductive MS-PCET reaction of hydrogenation of 2,4,6-^{t}Bu$_3$PhO$^{\bullet}$ between 0.25 mM **VW$_{12}$** and varied concentrations of [2,4,6-^{t}Bu$_3$PhO$^{\bullet}$]/[4-MeOPyrD^{+}(OTf^{-})] in MeCN at 20 °C with (gray) raw data and (red) fitting curve, along with fit-derived k_{obs}. 37

Figure S33. Plots of absorbance at 626 nm over time for the reductive MS-PCET reaction of hydrogenation of 2,4,6-^{t}Bu$_3$PhO$^{\bullet}$ between 0.25 mM **VW$_{12}$** and 3 mM [2,4,6-^{t}Bu$_3$PhO$^{\bullet}$]/[4-MeOPyrH^{+}(BF$_4^{-}$)] in MeCN at varied temperature between 0 and 10 °C with (gray) raw data and (red) fitting curve, along with fit-derived k_{obs}. 38

REFERENCES 39

General Considerations and Physical Measurements.

All the experiments were conducted in a UniLab MBraun inert atmosphere N_2 filled glove box. All glassware was oven-dried and cooled in an evacuated antechamber prior to use. All solvents were dried and deoxygenated on a solvent purification system (Pure Process Technology, LLC) and stored over activated 3 Å molecular sieves. The polyoxotungstate clusters $[^{n}Bu_4N]_3[VW_{12}O_{40}]$ (**VW_{12}**) and one-electron reduced $[^{n}Bu_4N]_4[VW_{12}O_{40}]$ (**$1e^{-}$-VW_{12}**), the organic radical 2,4,6-$^{t}Bu_3PhO^{\bullet}$, and deuterated 2,4,6-$^{t}Bu_3PhOD$ were synthesized according to the previous reports;[1–4] organic acids were synthesized via the reactions between the corresponding conjugated bases and $HBF_4{\bullet}Et_2O$ or trifluoromethanesulfonic acid-*d* (DOTf) according to the published method;[5] all other chemicals were used as received without special treatment.

NMR studies were carried out on a Brüker 400 MHz spectrometer and a Brüker 500 MHz spectrometer and recorded in MeCN-d_3 at room temperature. ^1H NMR was calibrated by using the solvent acetonitrile as 1.940 ppm. Electron paramagnetic resonance (EPR) studies were performed on a Brüker EMXplus EPR spectrometer at 10 K. Electronic absorption spectroscopy was recorded in dry acetonitrile in 1-cm-path quartz cuvettes with Agilent Cary 3500 Multicell UV-vis spectrophotometer.

***Kinetics analysis*.**

Pesudo-1st-order reaction conditions were used to find the rate expression for both oxidative and reductive reactions. The growth of absorbance at 400 nm of 2,4,6-$^{t}Bu_3PhO^{\bullet}$, 465 nm of Hantzsch pyridine (HE), and the decrease of absorbance at 626 nm 2,4,6-$^{t}Bu_3PhO^{\bullet}$ ($A_{400\ nm}$ of 2,4,6-$^{t}Bu_3PhO^{\bullet}$ is over instrument saturation due to the excess usage in the reductive MS-PCET hydrogenation) were tracked over time to determine the pseudo-first order rate constant, k_{obs}, through the following Equation S1:

$$A_t = A_{inf} + (A_0 - A_{inf})e^{k_{obs}t} \qquad \text{Eqn. S1}$$

Where A_t is the absorbance at a given time, t, in seconds; A_{inf} is the absorbance at the end of the reaction (t = infinite); A_0 is the initial absorbance; and k_{obs} is the observed 1st-order rate constant (s^{-1}). Error was determined by calculating the standard deviation of k_{obs} between triplicate trials. Eyring analysis was performed in similar manner to find the rate expression at varied temperatures. The Eyring analysis results are plotted in $\ln(k_{obs}/T)$ vs $(1/T)$, as follows in Equation S2. The Gibbs energy of activation is calculated by the following Equation S3.

$$\ln\left(\frac{k_{obs}}{T}\right) = \frac{\Delta H^{\ddagger}}{R} \times \frac{1}{T} + ln\frac{k_B}{h} + \frac{\Delta S^{\ddagger}}{R} \qquad \text{Eqn. S2}$$

$$\Delta G^{\ddagger} = \Delta H^{\ddagger} - T\Delta S^{\ddagger} \qquad \text{Eqn. S3}$$

Where T is the temperature in Kelvin, $\Delta H^{\ddagger}$ is the enthalpy of activation, R is gas constant, k_B is Boltzmann constant, h is Planck constant, $\Delta S^{\ddagger}$ is the entropy of activation, $\Delta G^{\ddagger}$ is the Gibbs energy of activation. To determine the deuterium kinetic isotope effect (KIE), analogous pseudo-first order reactions were performed under identical conditions, using the deuterium-labelled species 2,4,6-$^{t}Bu_3PhOD$ or 4-MeOPyrD^{+}OTf^{-}. The KIE is calculated according to Equation S4:

$$k_H/k_D = \text{KIE} \qquad \text{Eqn. S4}$$

Table S1. Brønsted bases used in this work.[6]

Chemical Structure	Name	Abbreviation	pK_{aH} in MeCN
Primary amine			
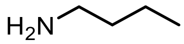	*n*-butylamine	$^{n}BuNH_2$	18.26
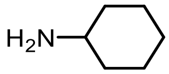	cyclohexylamine	CHA	18.36
Secondary amine			
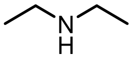	diethylamine	Et_2NH	18.75
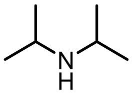	diisopropylamine	$^{i}Pr_2NH$	18.82
Tertiary amine			
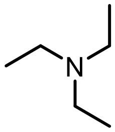	triethylamine	Et_3N	18.83
Cyclic amine			
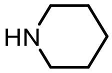	piperidine		19.35
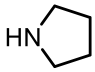	pyrrolidine		19.62
Heterocycle			
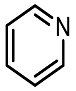	pyridine	Pyr	12.53
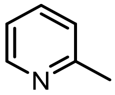	2-picoline	2-Pic	13.28
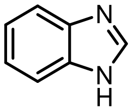	benzimidazole	BimH	13.54
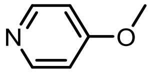	4-methoxylpyridine	4-MeOPyr	14.24
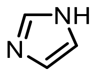	imidazole	ImH	15.07
Other			
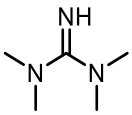	1,1,3,3-tetramethylguanidine	TMG	23.35

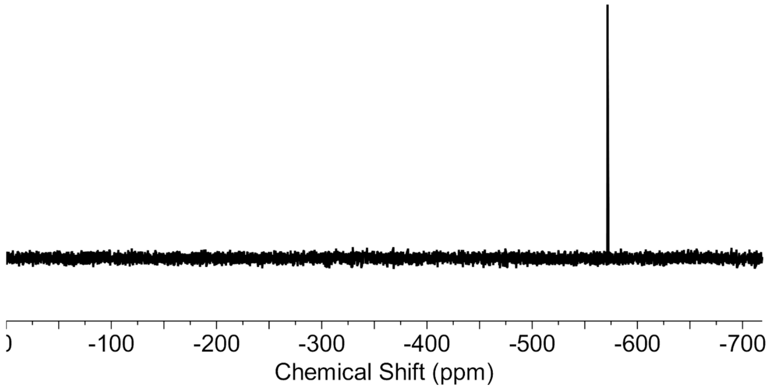

Figure S1. ^{51}V NMR spectrum of equimolar **VW$_{12}$** and TMG in MeCN-d_3, showing the stability of **VW$_{12}$** in the presence of a strong organic base.

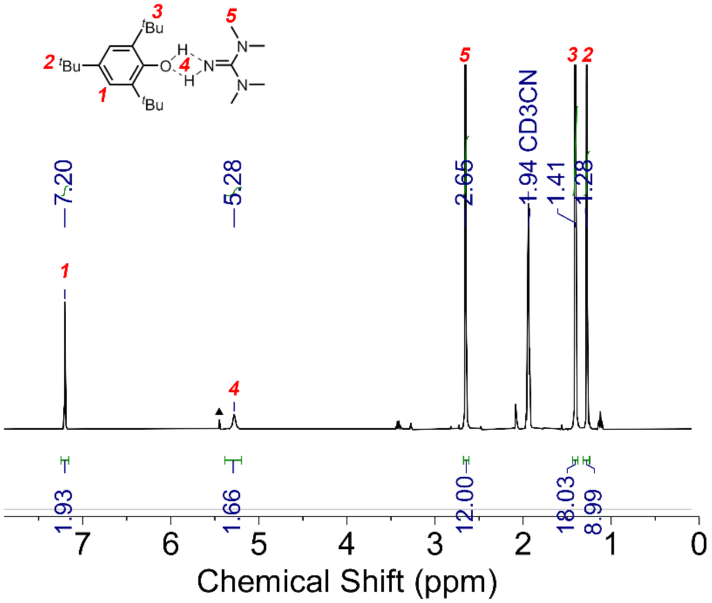

Figure S2. ^{1}H NMR spectrum of equimolar TTBP and TMG in MeCN-d_3, showing the formation of hydrogen-bonded adduct.

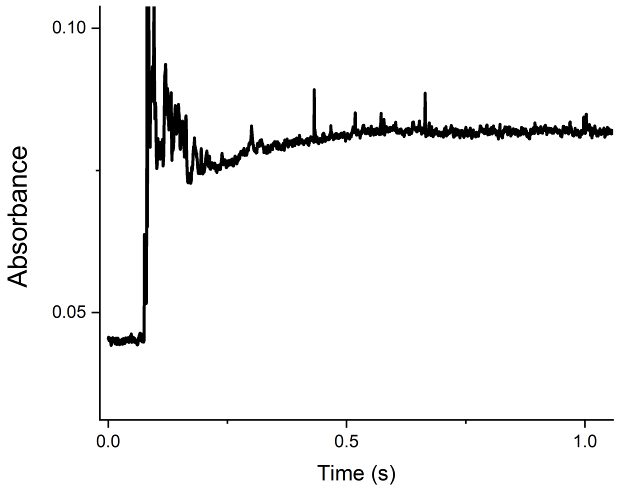

Figure S3. Plot of absorbance at 400 nm over time for the oxidative MS-PCET reaction of dehydrogenation of TTBP between 0.02 mM $\mathbf{VW_{12}}$ and 2 mM [TTBP]/[TMG] in MeCN at −30 °C

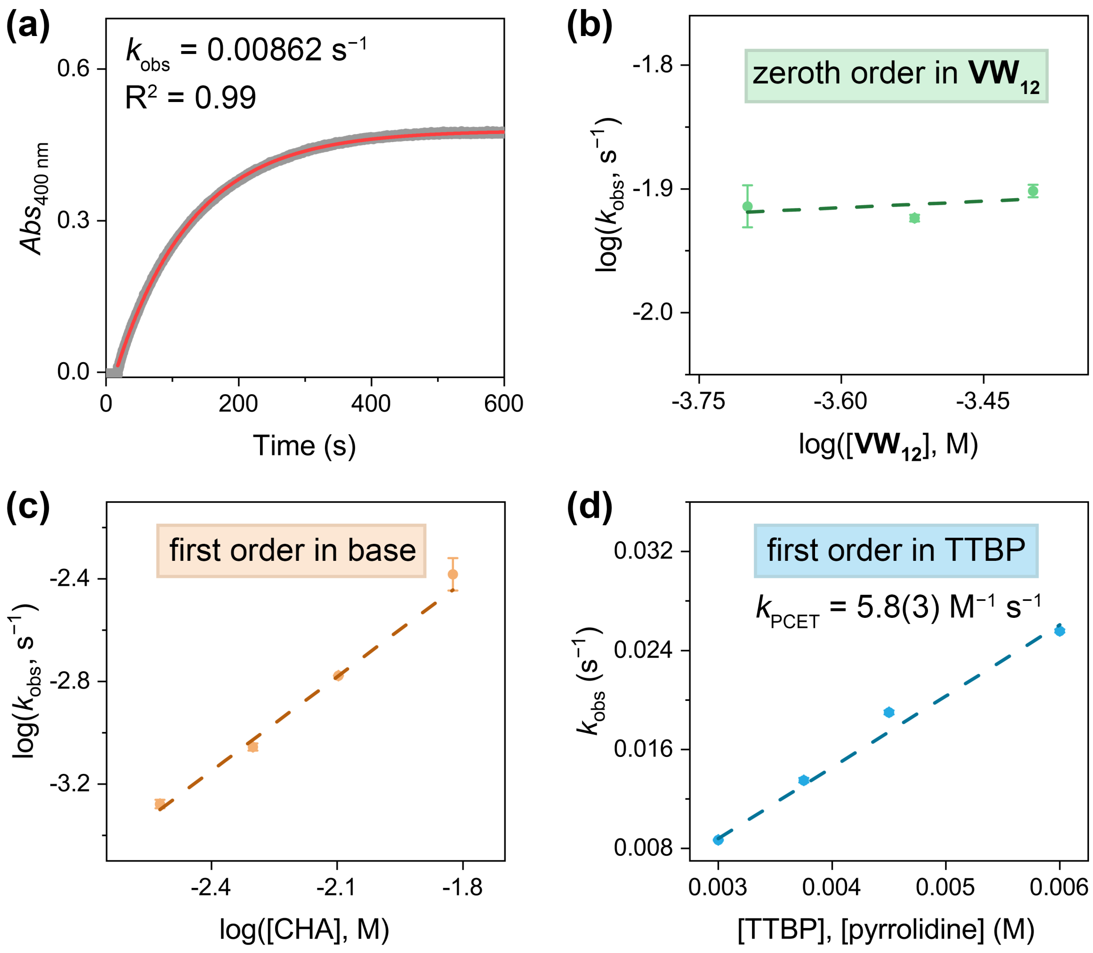

Figure S4. (a) Determination of the observed pseudo-first-order rate constant for the oxidative MS-PCET reaction of TTBP (3 mM) by $\mathbf{VW_{12}}$ (0.25 mM)/pyrrolidine (3 mM) in MeCN at 20 °C by monitoring the absorbance change at 400 nm over the time. Gray dots are raw absorbance data points and the red line is the single exponential fitted trace. (b) Plot of log(k_{obs}) with respect to log([$\mathbf{VW_{12}}$]). The concentrations of [TTBP] and [pyrrolidine] are held constant at 4.5 mM. (c) Plot of log(k_{obs}) with respect to log([CHA]). The concentrations of [$\mathbf{VW_{12}}$] and [TTBP] are held constant at 0.25 and 3 mM, respectively. (d) Plot of k_{obs} as the function of [TTBP] and [pyrrolidine]. The concentration of [$\mathbf{VW_{12}}$] is held constant at 0.25 mM.

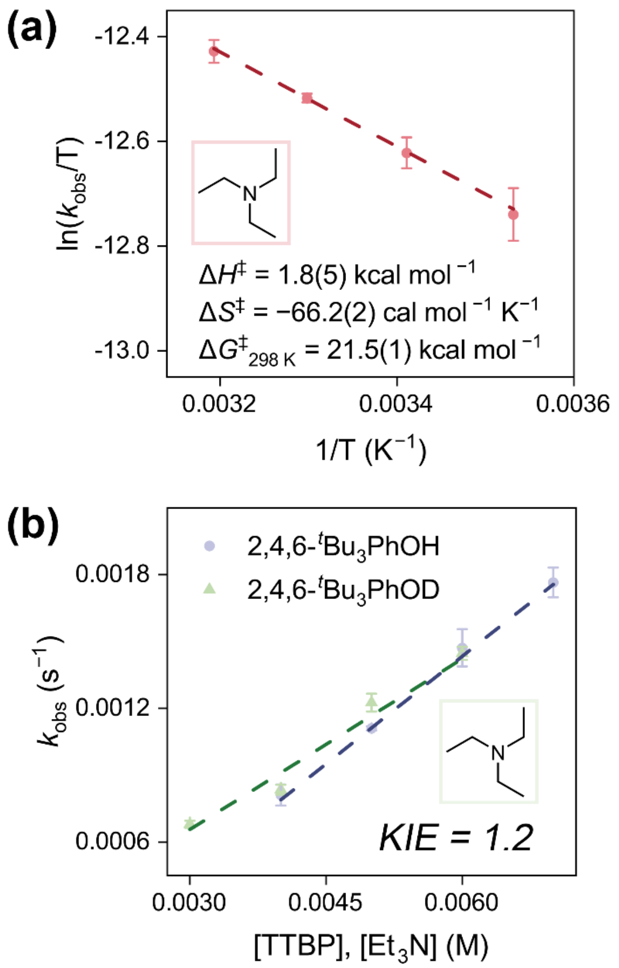

Figure S5. (a) Eyring plots of the MS-PCET dehydrogenation reaction of TTBP in MeCN by 0.25 mM $\mathbf{VW_{12}}$/5 mM Et_3N between 10 and 40 °C. (b) Plots of k_{obs} with respect to the concentrations of [2,4,6-$^tBu_3PhOH$] or [2,4,6-$^tBu_3PhOD$] with [Et_3N] at 30 °C, showing the KIE value of 1.2. The concentration of [$\mathbf{VW_{12}}$] was held constant at 0.25 mM.

[TTBP] = [^{n}BuNH$_2$] = 3 mM

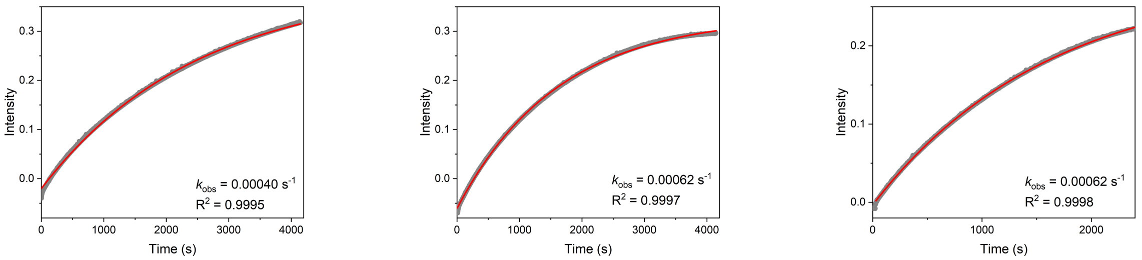

[TTBP] = [^{n}BuNH$_2$] = 4.5 mM

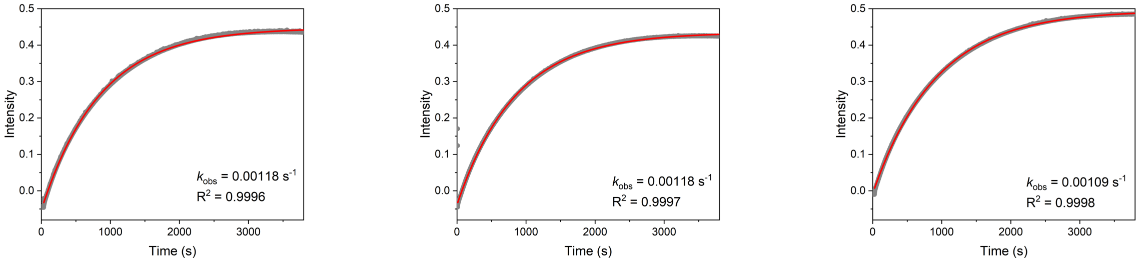

[TTBP] = [^{n}BuNH$_2$] = 6 mM

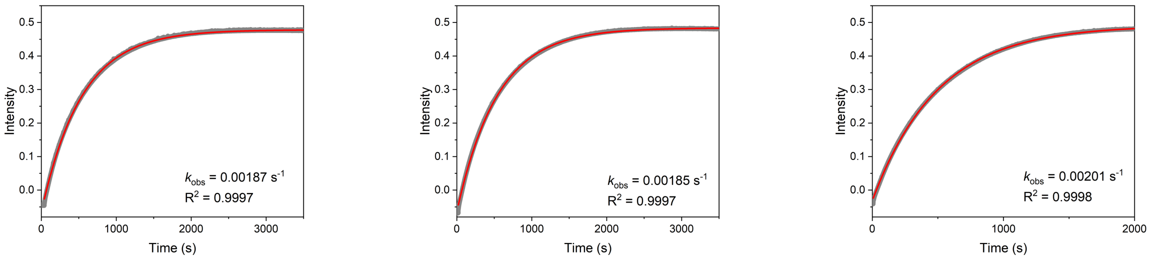

Figure S6. Plots of absorbance at 400 nm over time for the oxidative MS-PCET reaction of dehydrogenation of TTBP between 0.25 mM **VW$_{12}$** and varied concentrations of [TTBP]/[^{n}BuNH$_2$] in MeCN at 20 °C with (gray) raw data and (red) fitting curve, along with fit-derived k_{obs}.

[TTBP] = [CHA] = 3 mM

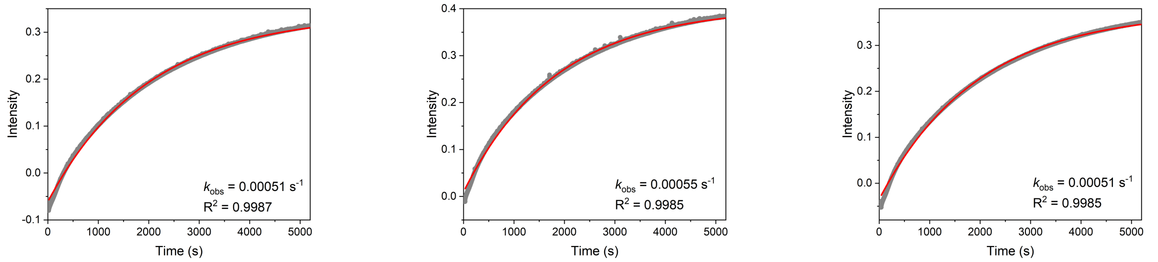

[TTBP] = [CHA] = 3.75 mM

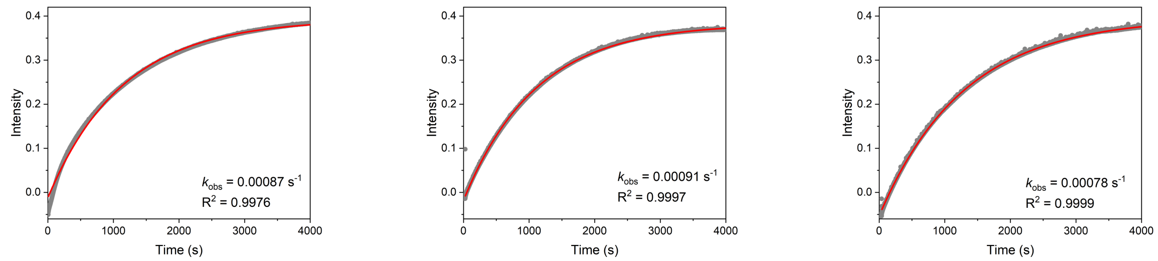

[TTBP] = [CHA] = 4.5 mM

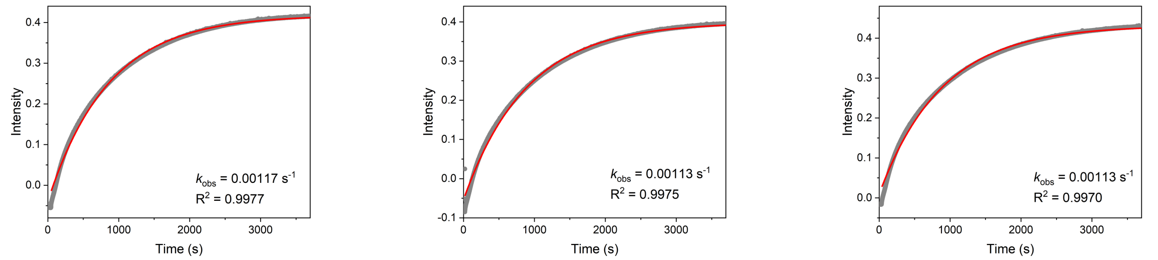

[TTBP] = [CHA] = 6 mM

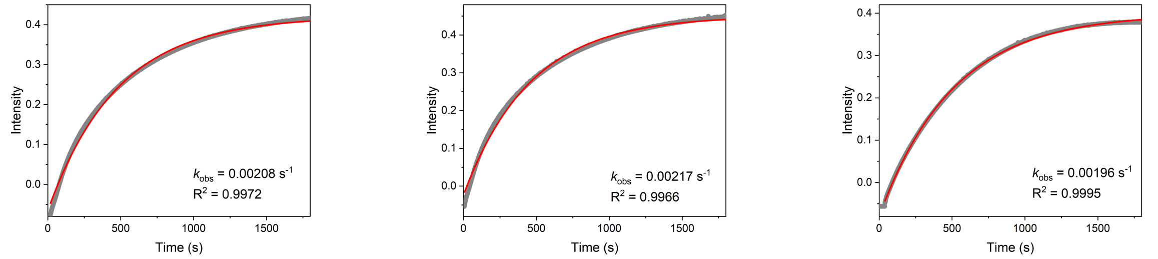

Figure S7. Plots of absorbance at 400 nm over time for the oxidative MS-PCET reaction of dehydrogenation of TTBP between 0.25 mM $\mathbf{VW_{12}}$ and varied concentrations of [TTBP]/[CHA] in MeCN at 20 °C with (gray) raw data and (red) fitting curve, along with fit-derived k_{obs}.

[TTBP] = [Et_2NH] = 3 mM

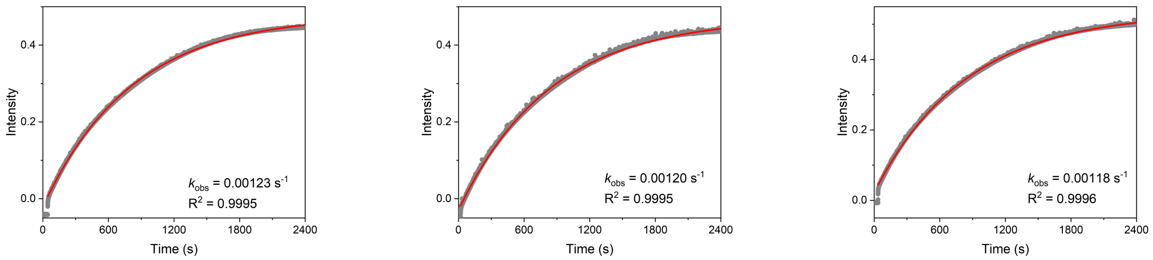

[TTBP] = [Et_2NH] = 3.75 mM

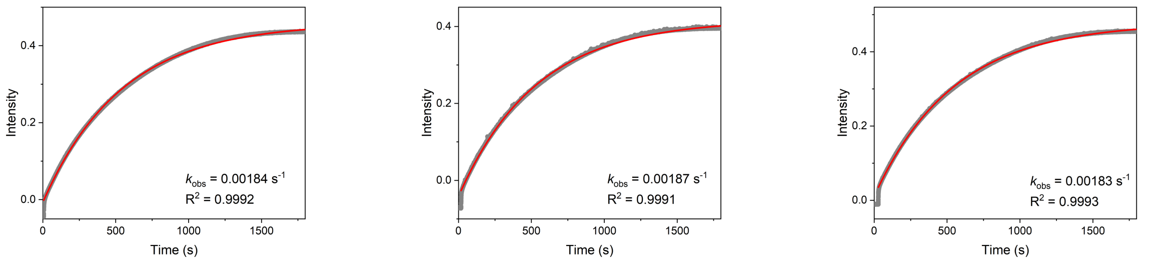

[TTBP] = [Et_2NH] = 4.5 mM

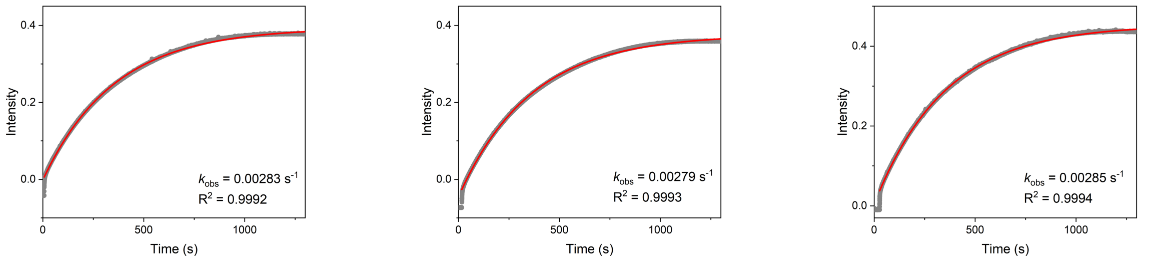

[TTBP] = [Et_2NH] = 6 mM

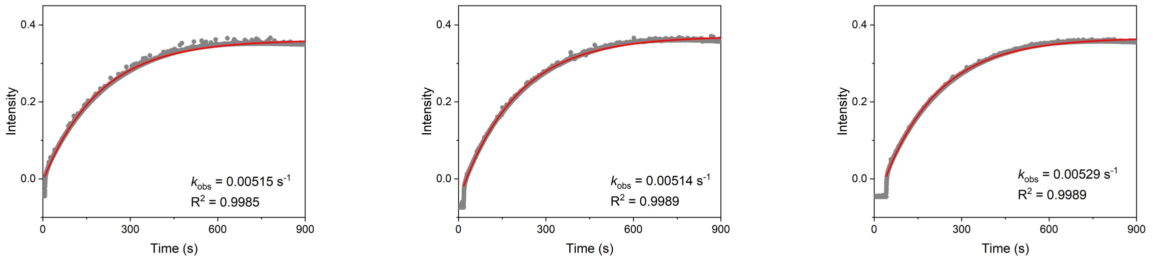

Figure S8. Plots of absorbance at 400 nm over time for the oxidative MS-PCET reaction of dehydrogenation of TTBP between 0.25 mM $\mathbf{VW_{12}}$ and varied concentrations of [TTBP]/[Et_2NH] in MeCN at 20 °C with (gray) raw data and (red) fitting curve, along with fit-derived k_{obs}.

[TTBP] = [^{i}Pr$_2$NH] = 3.75 mM

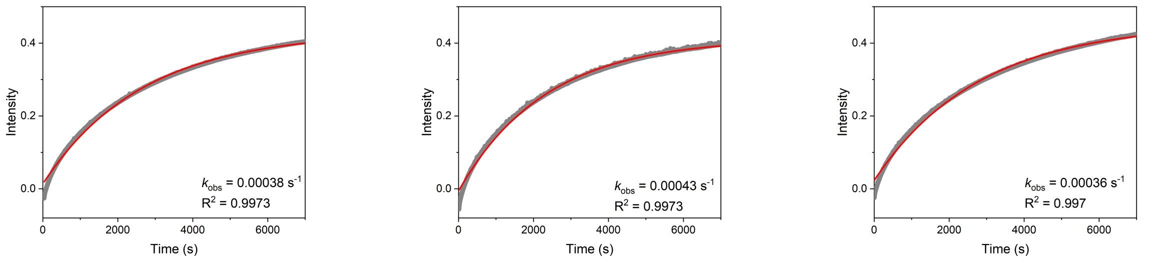

[TTBP] = [^{i}Pr$_2$NH] = 4.5 mM

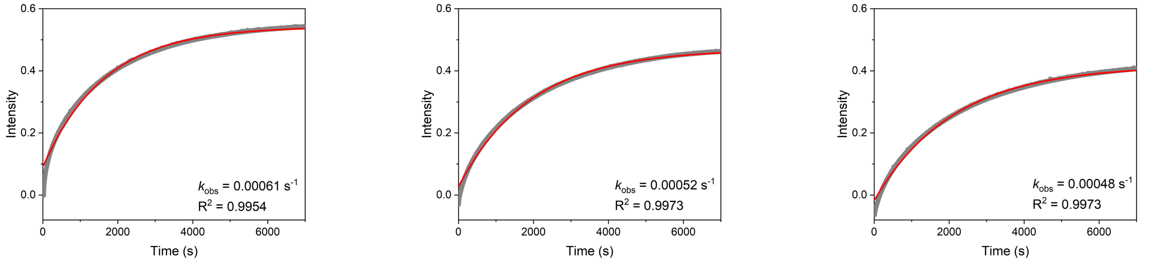

[TTBP] = [^{i}Pr$_2$NH] = 6 mM

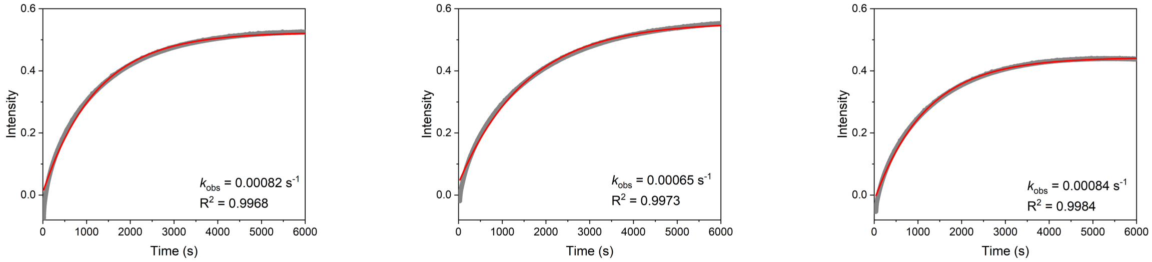

Figure S9. Plots of absorbance at 400 nm over time for the oxidative MS-PCET reaction of dehydrogenation of TTBP between 0.25 mM **VW$_{12}$** and varied concentrations of [TTBP]/[^{i}Pr$_2$NH] in MeCN at 20 °C with (gray) raw data and (red) fitting curve, along with fit-derived k_{obs}.

[TTBP] = [Et_3N] = 3 mM

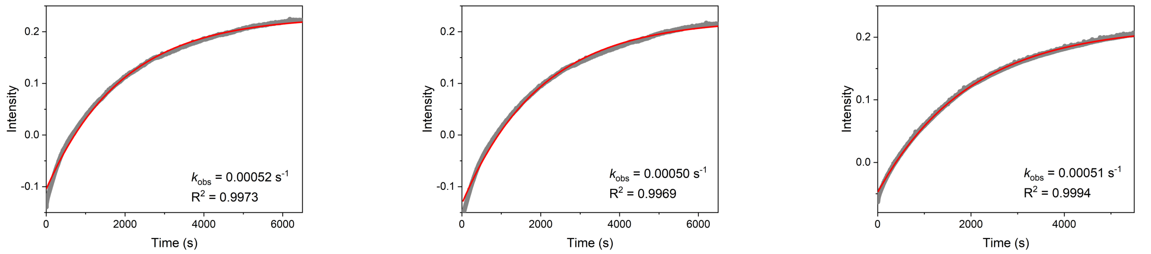

[TTBP] = [Et_3N] = 3.75 mM

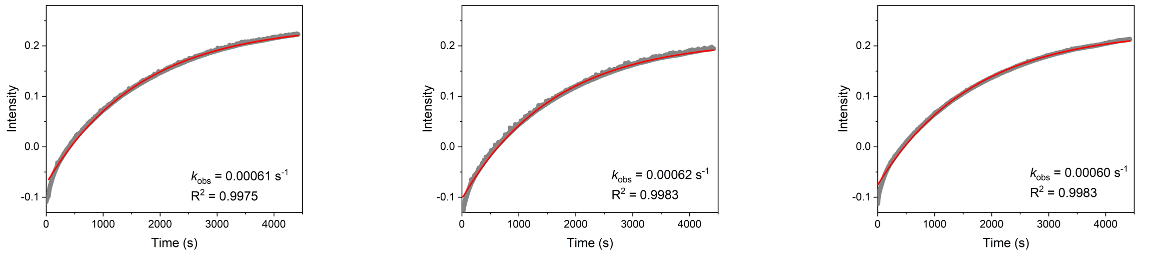

[TTBP] = [Et_3N] = 4.5 mM

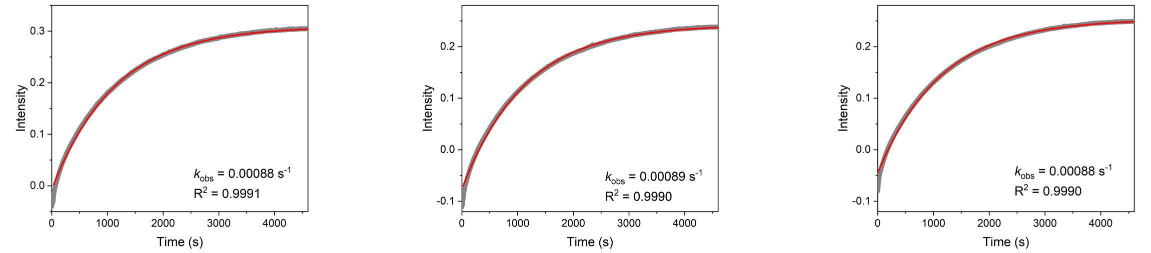

[TTBP] = [Et_3N] = 5 mM

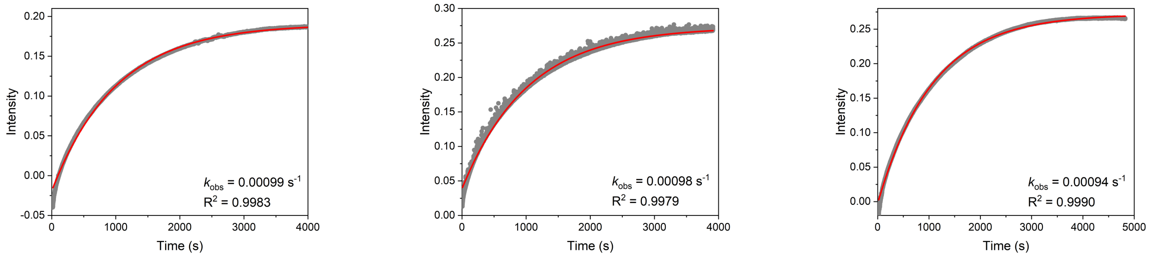

[TTBP] = [Et_3N] = 6 mM

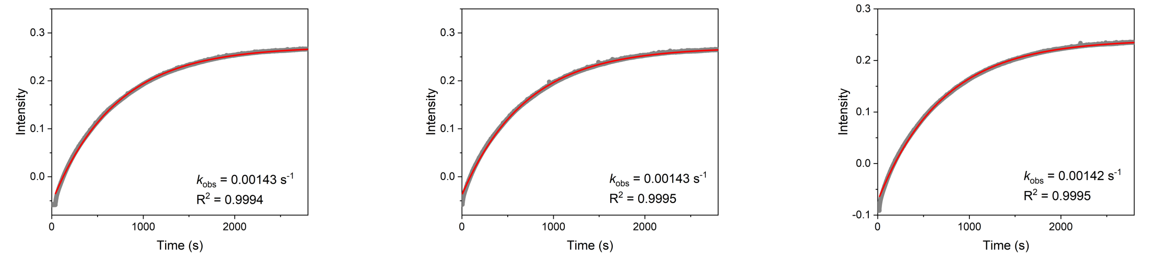

Figure S10. Plots of absorbance at 400 nm over time for the oxidative MS-PCET reaction of dehydrogenation of TTBP between 0.25 mM $\mathbf{VW_{12}}$ and varied concentrations of [TTBP]/[Et_3N] in MeCN at 20 °C with (gray) raw data and (red) fitting curve, along with fit-derived k_{obs}.

[TTBP] = [piperidine] = 3 mM

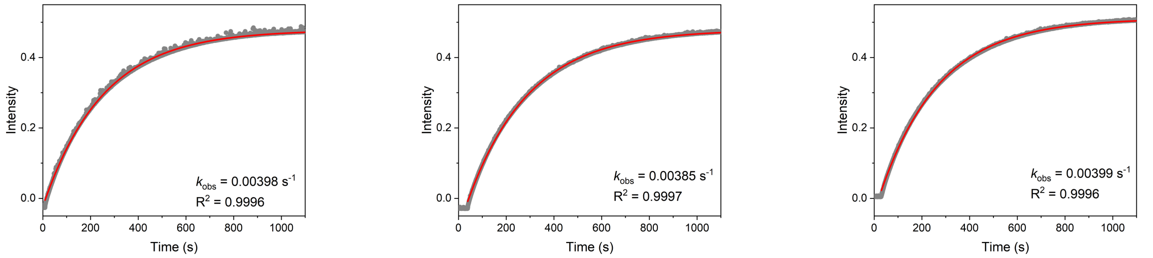

[TTBP] = [piperidine] = 3.75 mM

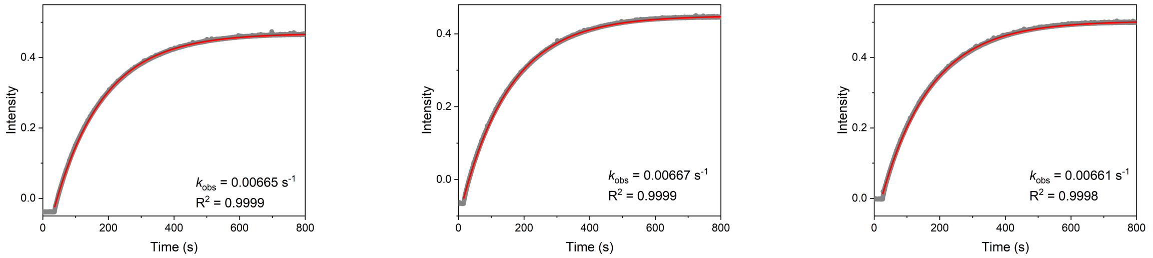

[TTBP] = [piperidine] = 4.5 mM

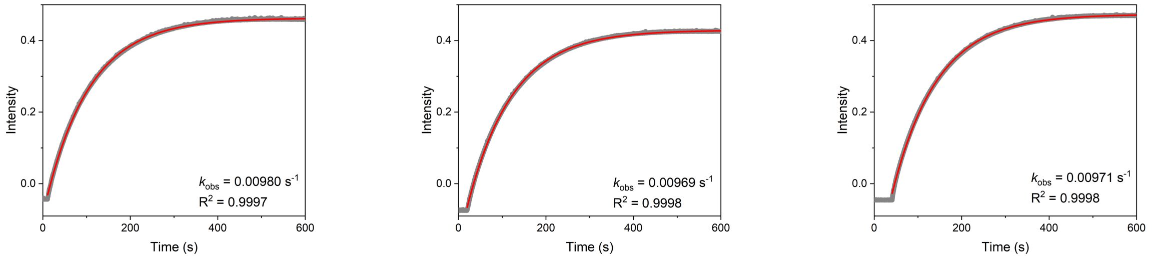

[TTBP] = [piperidine] = 6 mM

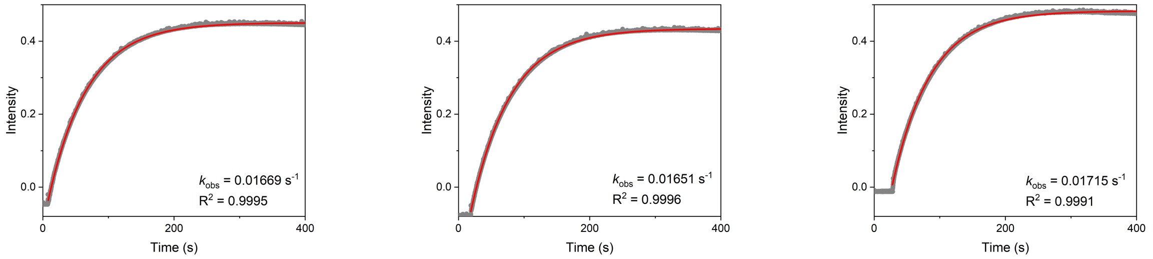

Figure S11. Plots of absorbance at 400 nm over time for the oxidative MS-PCET reaction of dehydrogenation of TTBP between 0.25 mM $\mathbf{VW_{12}}$ and varied concentrations of [TTBP]/[piperidine] in MeCN at 20 °C with (gray) raw data and (red) fitting curve, along with fit-derived k_{obs}.

[TTBP] = [pyrrolidine] = 3 mM

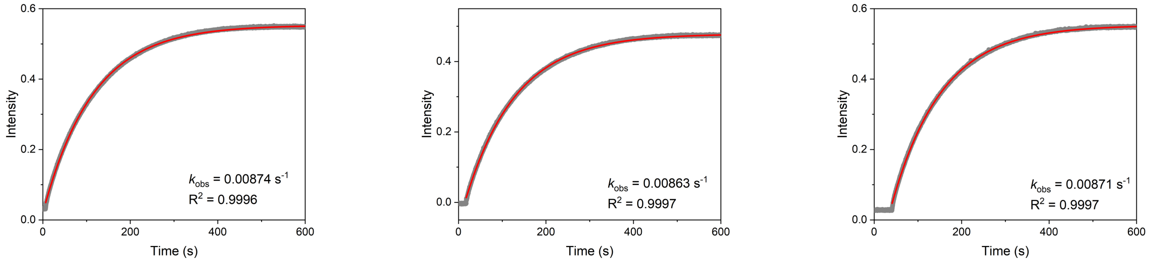

[TTBP] = [pyrrolidine] = 3.75 mM

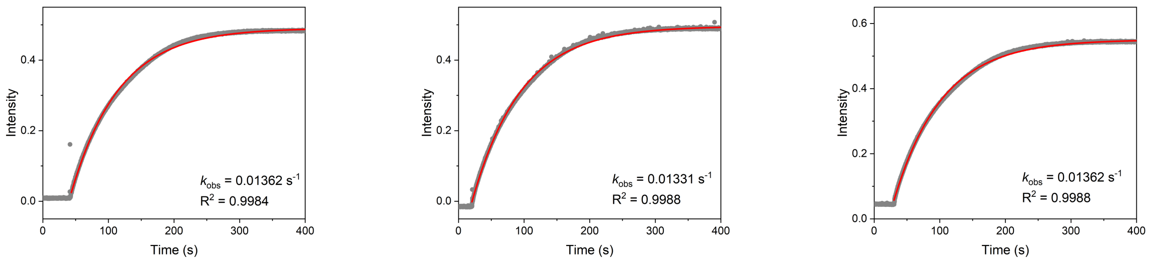

[TTBP] = [pyrrolidine] = 4.5 mM

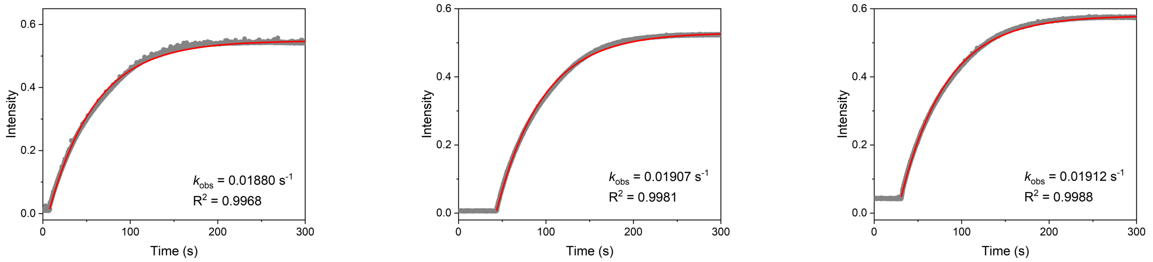

[TTBP] = [pyrrolidine] = 6 mM

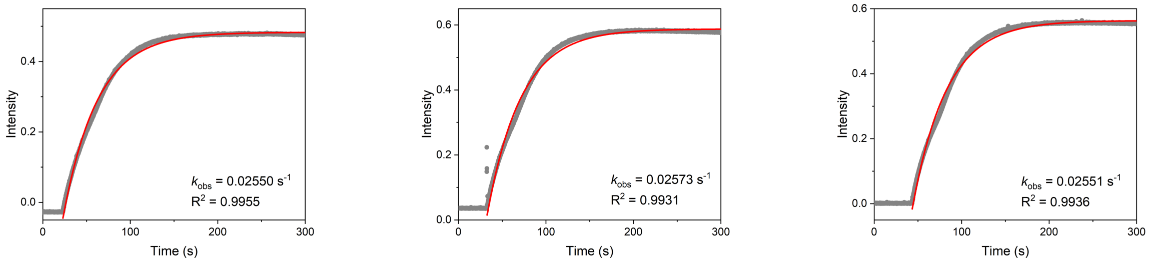

Figure S12. Plots of absorbance at 400 nm over time for the oxidative MS-PCET reaction of dehydrogenation of TTBP between 0.25 mM $\mathbf{VW_{12}}$ and varied concentrations of [TTBP]/[pyrrolidine] in MeCN at 20 °C with (gray) raw data and (red) fitting curve, along with fit-derived k_{obs}.

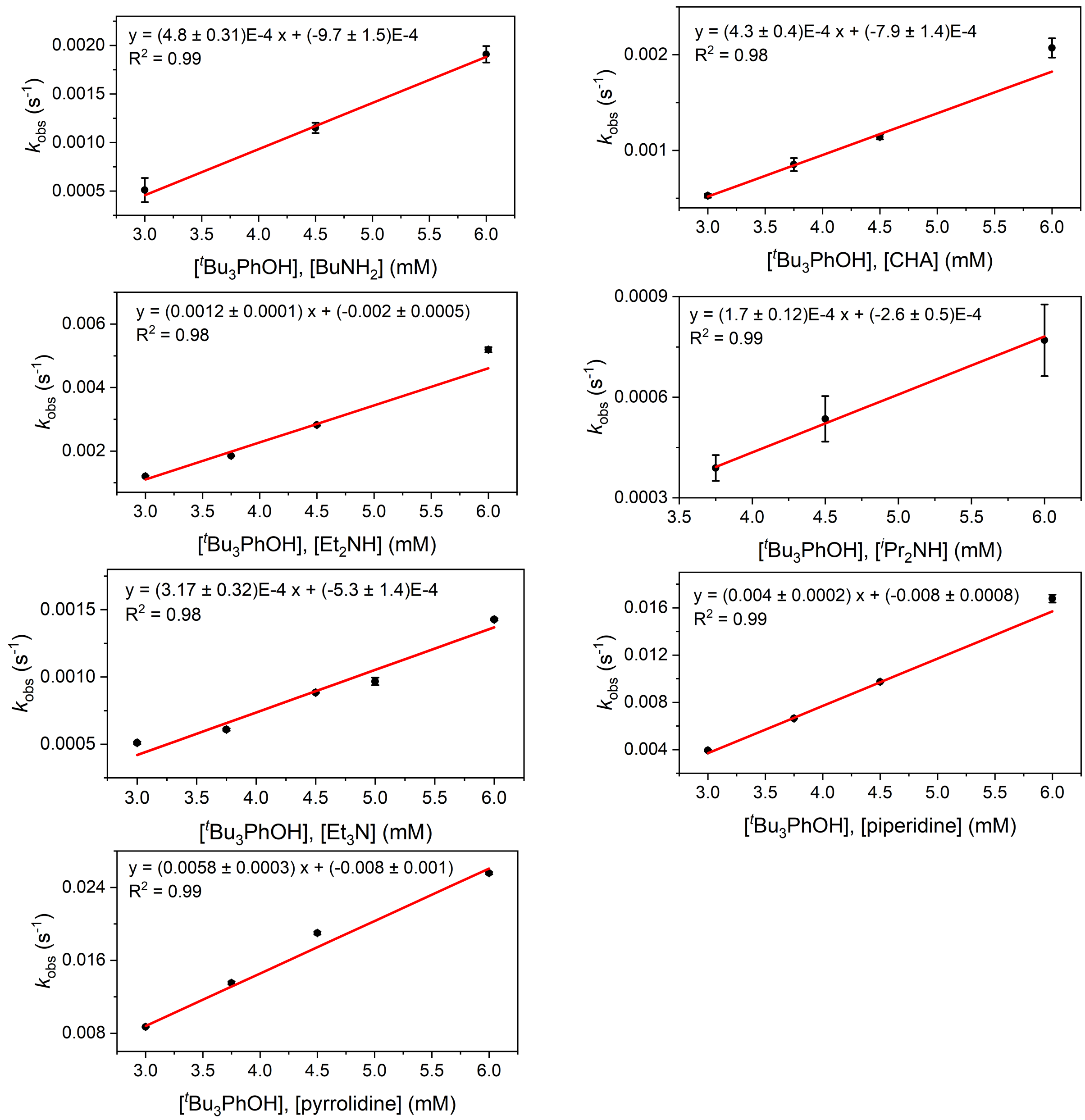

Figure S13. Plots of k_{obs} versus the concentrations of TTBP and different bases.

[**VW_{12}**] = 0.2 mM

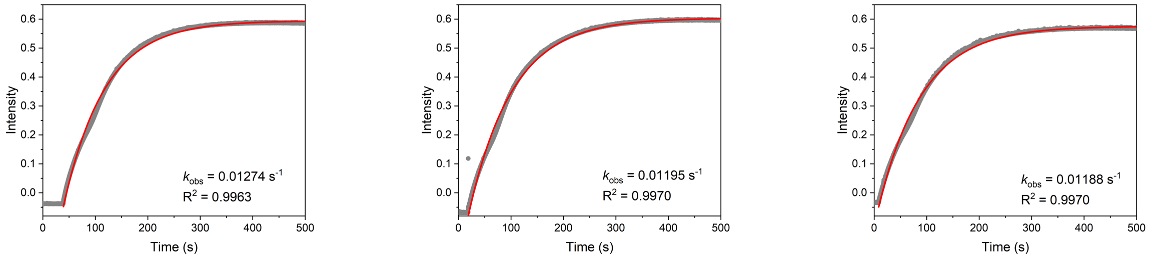

[**VW_{12}**] = 0.3 mM

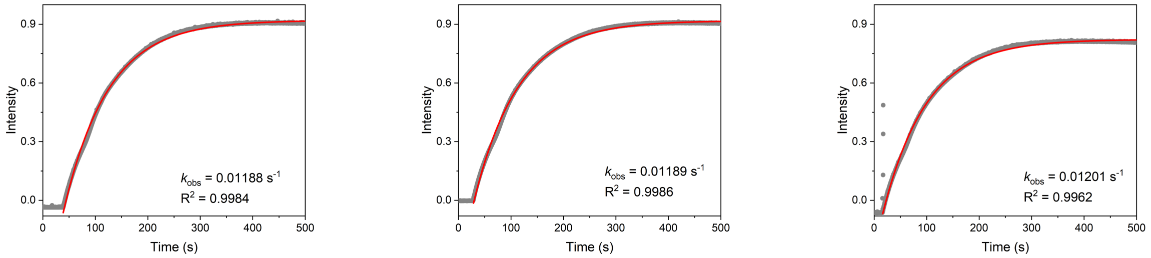

[**VW_{12}**] = 0.4 mM

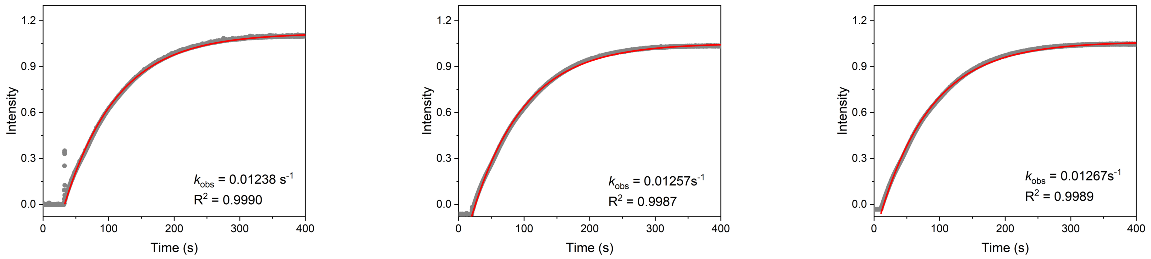

Figure S14. Plots of absorbance at 400 nm over time for the oxidative MS-PCET reaction of dehydrogenation of TTBP between varied concentrations of **VW_{12}** and 4.5 mM [TTBP]/[pyrrolidine] in MeCN at 20 °C with (gray) raw data and (red) fitting curve, along with fit-derived k_{obs}.

[CHA] = 3 mM

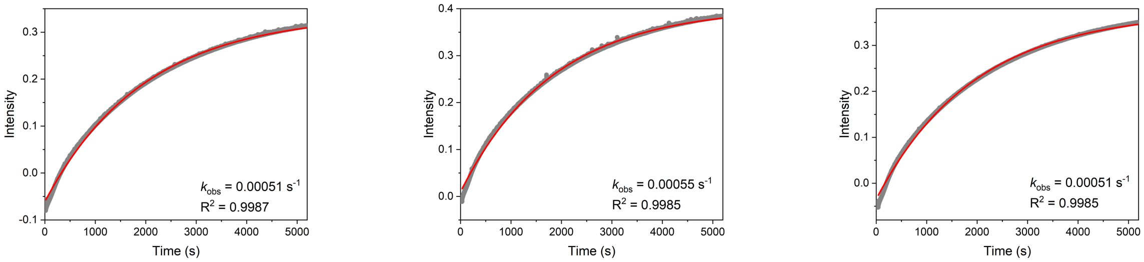

[CHA] = 5 mM

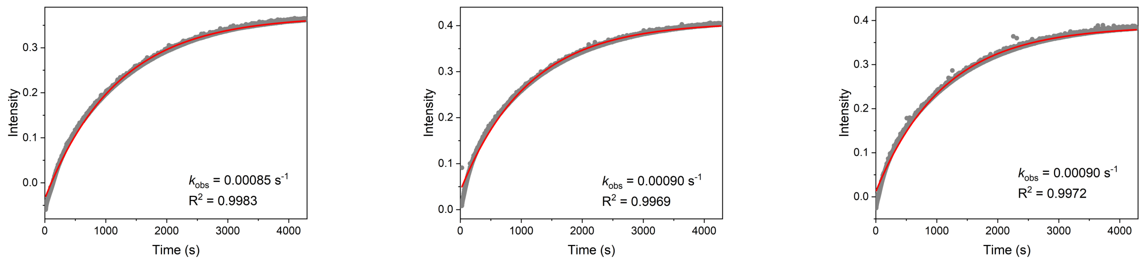

[CHA] = 8 mM

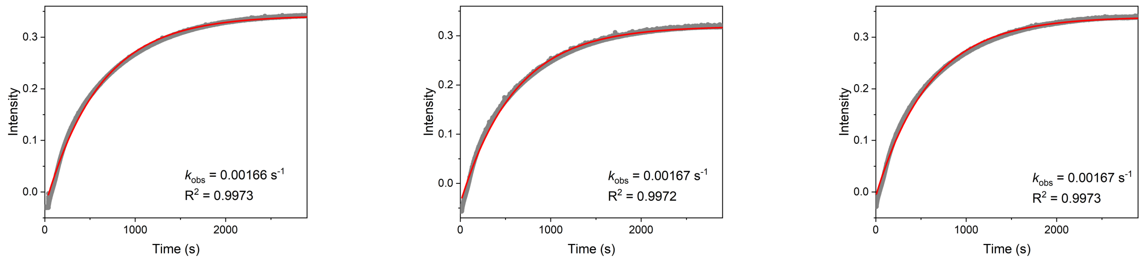

[CHA] = 15 mM

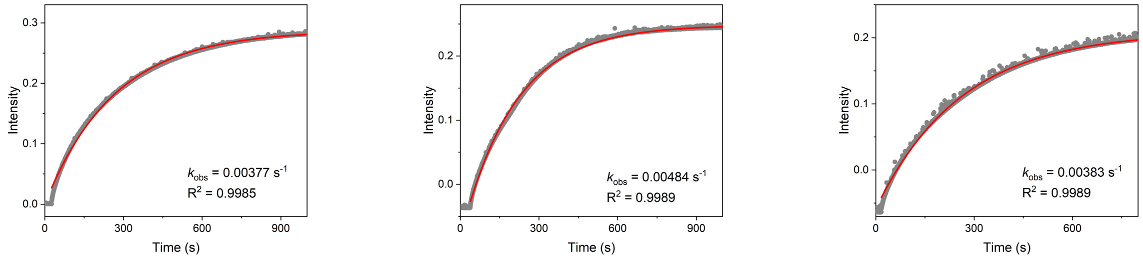

Figure S15. Plots of absorbance at 400 nm over time for the oxidative MS-PCET reaction of dehydrogenation of TTBP between 0.25 mM **VW$_{12}$**, 3 mM TTBP, and varied concentrations of [CHA] in MeCN at 20 °C with (gray) raw data and (red) fitting curve, along with fit-derived k_{obs}.

Temperature = −20 °C

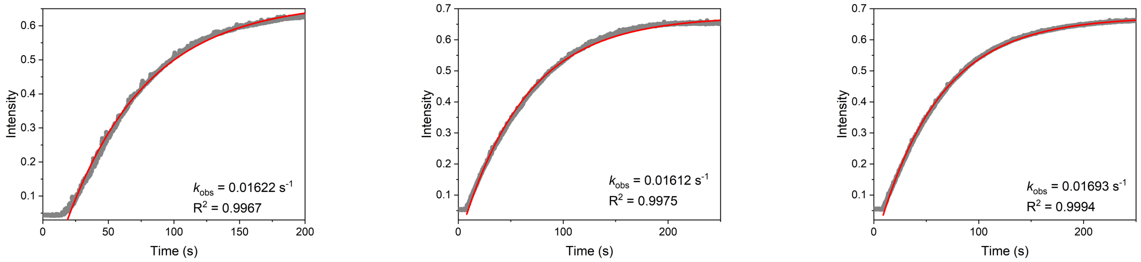

Temperature = 0 °C

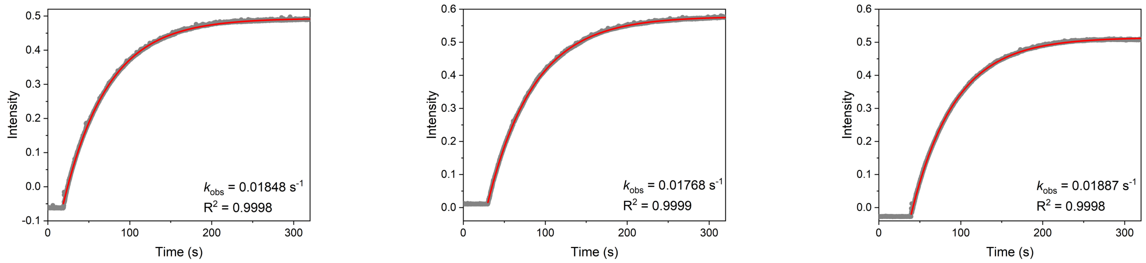

Temperature = 10 °C

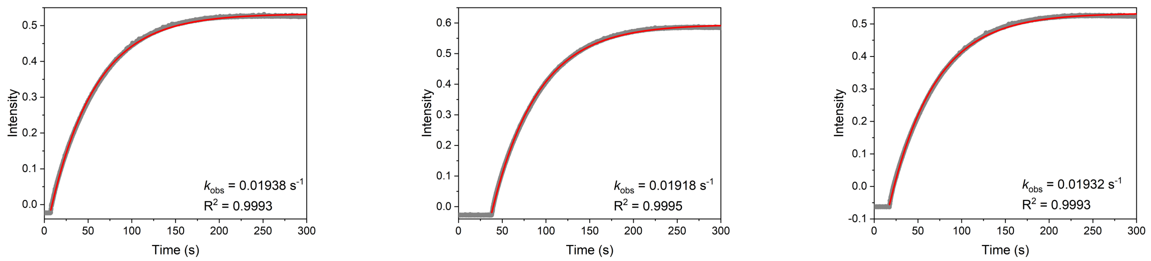

Temperature = 30 °C

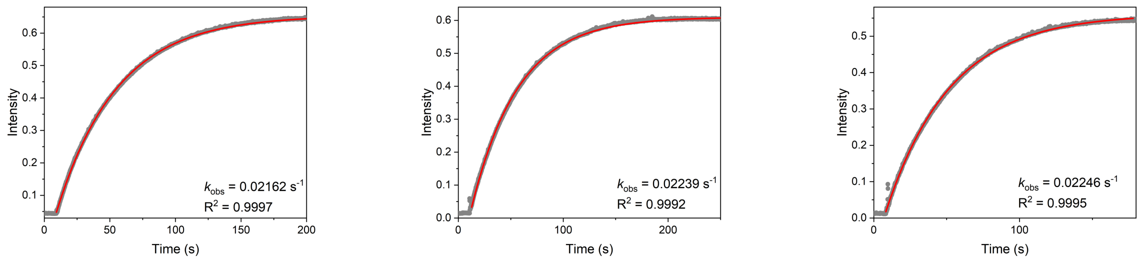

Figure S16. Plots of absorbance at 400 nm over time for the oxidative MS-PCET reaction of dehydrogenation of TTBP between 0.25 mM **VW$_{12}$** and 4.5 mM [TTBP]/[pyrrolidine] in MeCN at varied temperature between −20 and 30 °C with (gray) raw data and (red) fitting curve, along with fit-derived k_{obs}.

Temperature = 10 °C

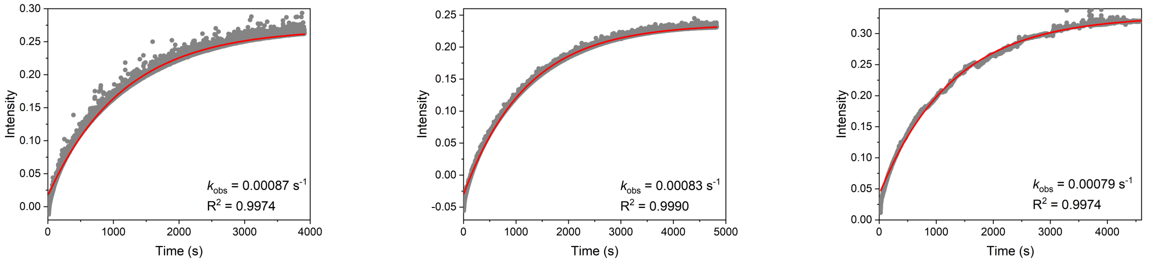

Temperature = 20 °C

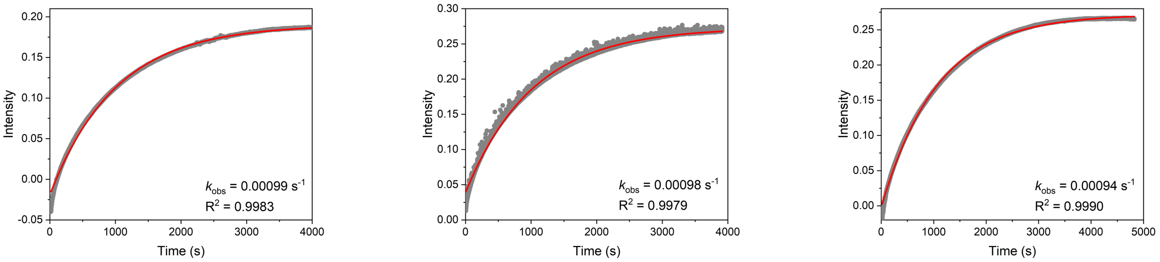

Temperature = 30 °C

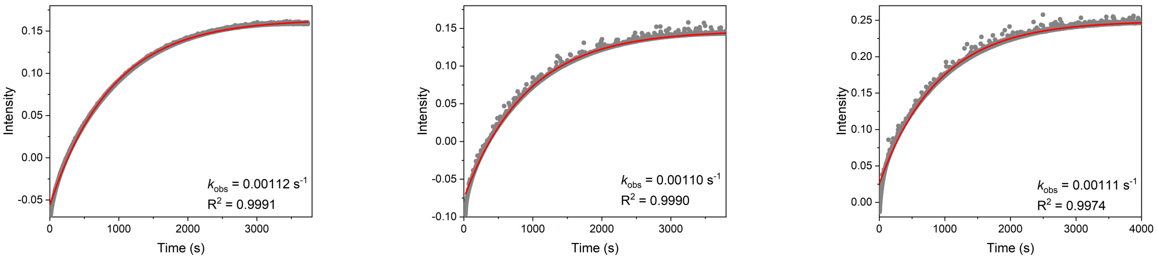

Temperature = 40 °C

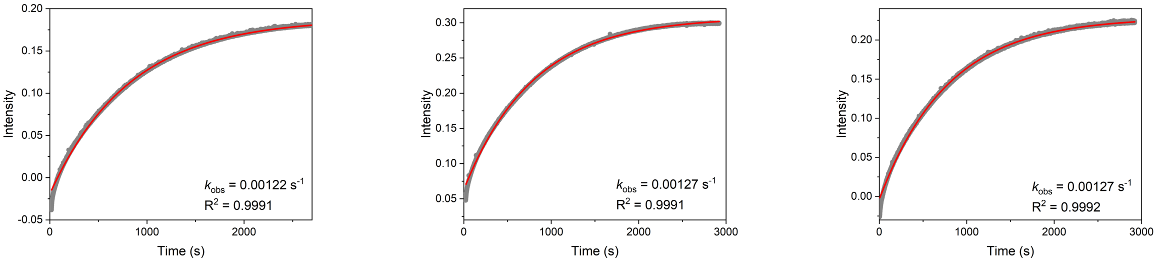

Figure S17. Plots of absorbance at 400 nm over time for the oxidative MS-PCET reaction of dehydrogenation of TTBP between 0.25 mM **VW_{12}** and 5 mM [TTBP]/[Et_3N] in MeCN at varied temperature between 10 and 40 °C with (gray) raw data and (red) fitting curve, along with fit-derived k_{obs}.

[2,4,6-^{t}Bu$_3$PhOD] = [CHA] = 3 mM

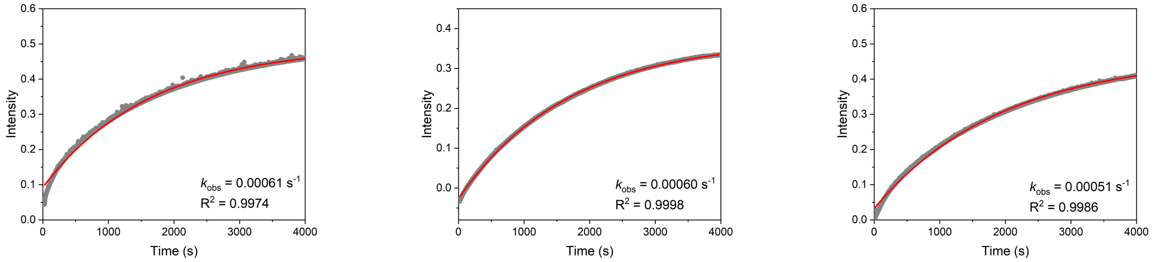

[2,4,6-^{t}Bu$_3$PhOD] = [CHA] = 3.75 mM

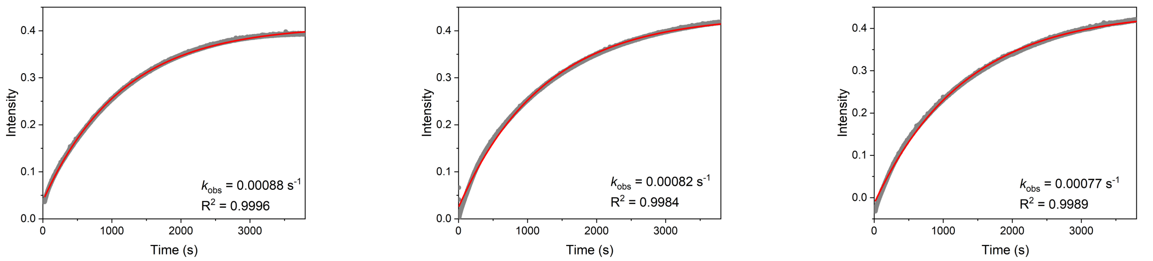

[2,4,6-^{t}Bu$_3$PhOD] = [CHA] = 4.5 mM

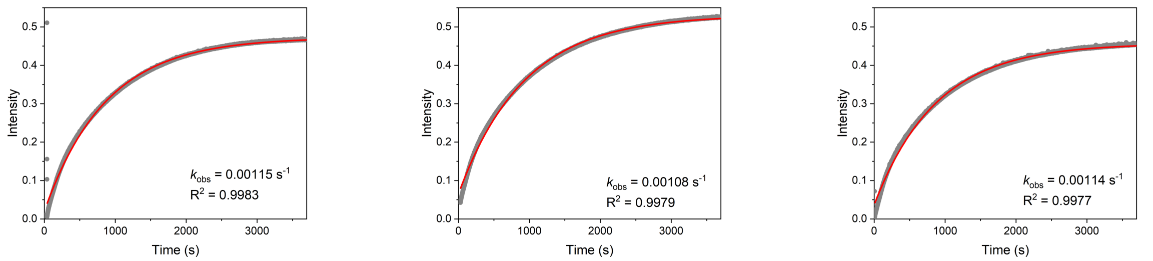

Figure S18. Plots of absorbance at 400 nm over time for the oxidative MS-PCET reaction of dehydrogenation of 2,4,6-^{t}Bu$_3$PhOD between 0.25 mM **VW$_{12}$** and varied concentrations of [2,4,6-^{t}Bu$_3$PHOD]/[pyrrolidine] in MeCN at 20 °C with (gray) raw data and (red) fitting curve, along with fit-derived k_{obs}.

[2,4,6-^{t}Bu$_3$PhOD] = [Et_3N] = 3 mM

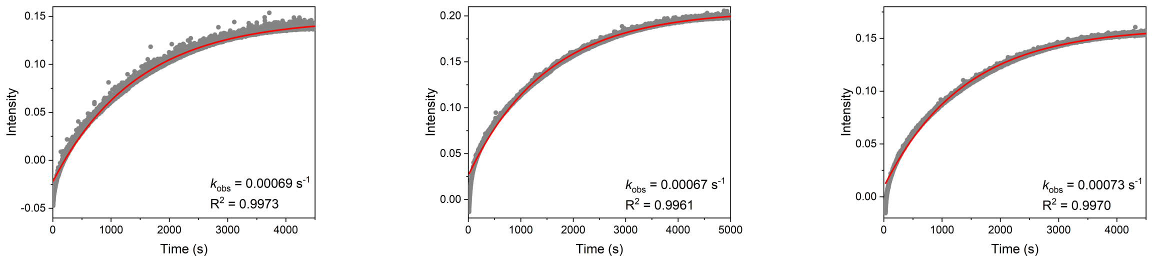

[2,4,6-^{t}Bu$_3$PhOD] = [Et_3N] = 4 mM

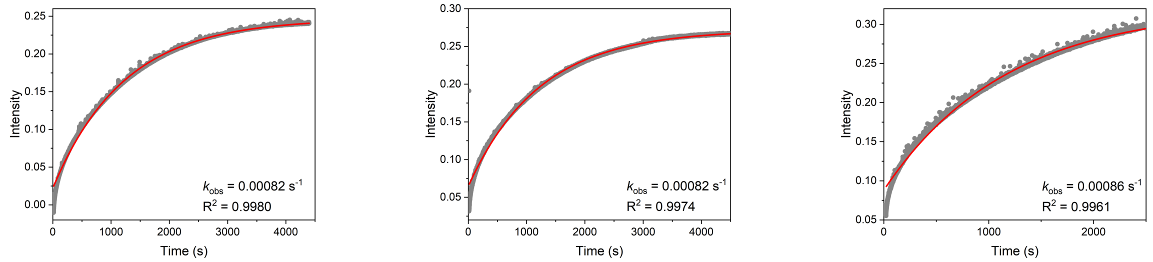

[2,4,6-^{t}Bu$_3$PhOD] = [Et_3N] = 5 mM

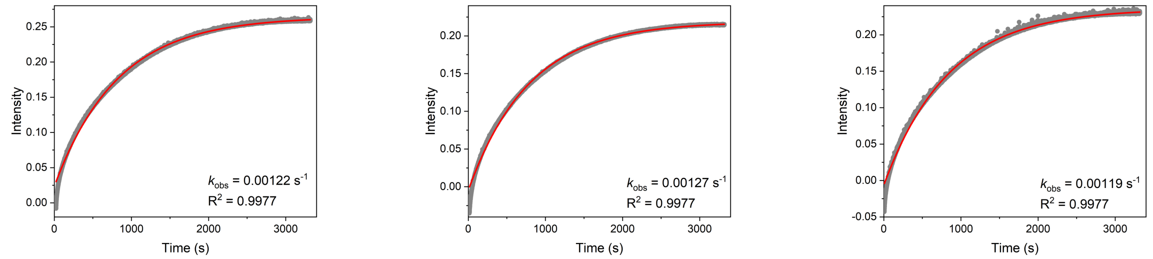

[2,4,6-^{t}Bu$_3$PhOD] = [Et_3N] = 6 mM

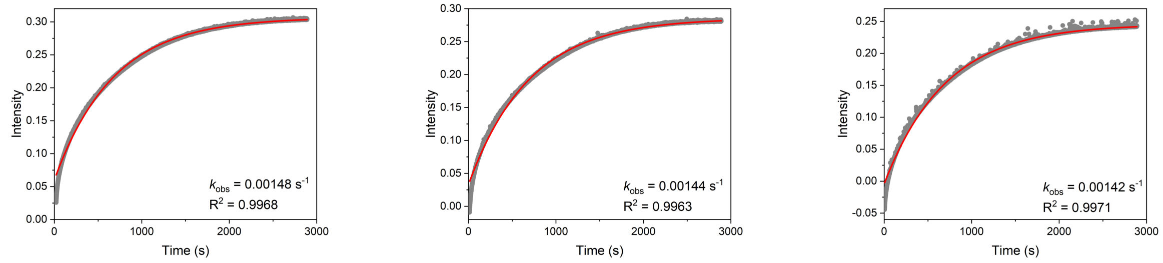

Figure S19. Plots of absorbance at 400 nm over time for the oxidative MS-PCET reaction of dehydrogenation of 2,4,6-^{t}Bu$_3$PhOD between 0.25 mM **VW$_{12}$** and varied concentrations of [2,4,6-^{t}Bu$_3$PhOD]/[Et_3N] in MeCN at 30 °C with (gray) raw data and (red) fitting curve, along with fit-derived k_{obs}.

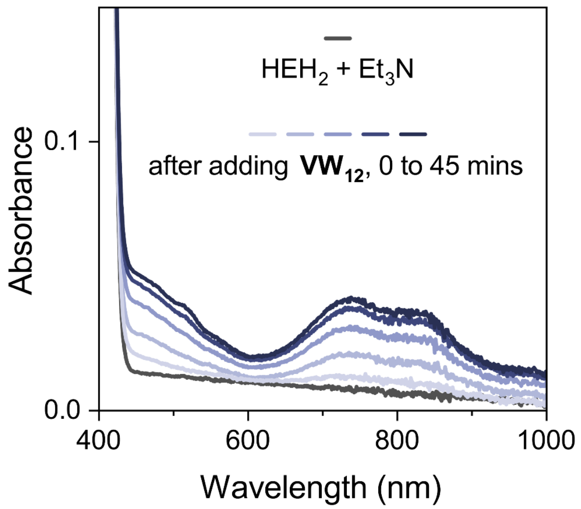

Figure S20. Electronic absorption spectra of 0.5 equiv Hantzsch ester (HEH_2) and 1 equiv Et_3N before and after the addition of 1 equiv $\mathbf{VW_{12}}$ over the time from 0 to 45 minutes.

[HEH$_2$] = [Et$_3$N] = 3 mM

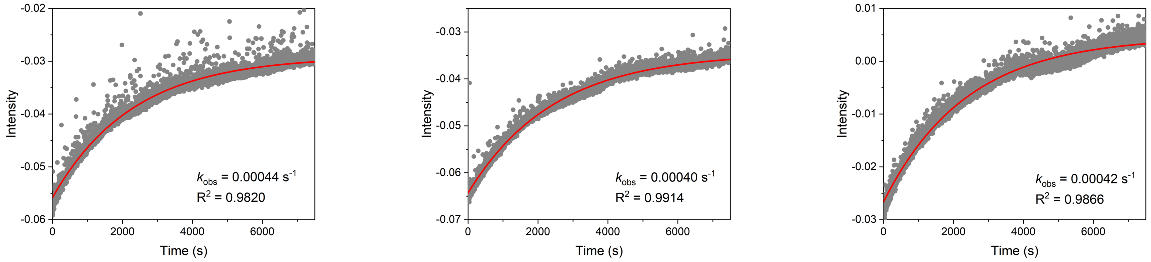

[HEH$_2$] = [Et$_3$N] = 3.75 mM

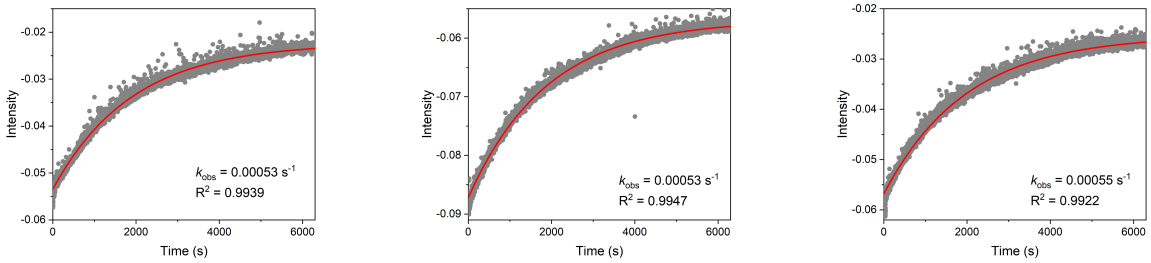

[HEH$_2$] = [Et$_3$N] = 4.5 mM

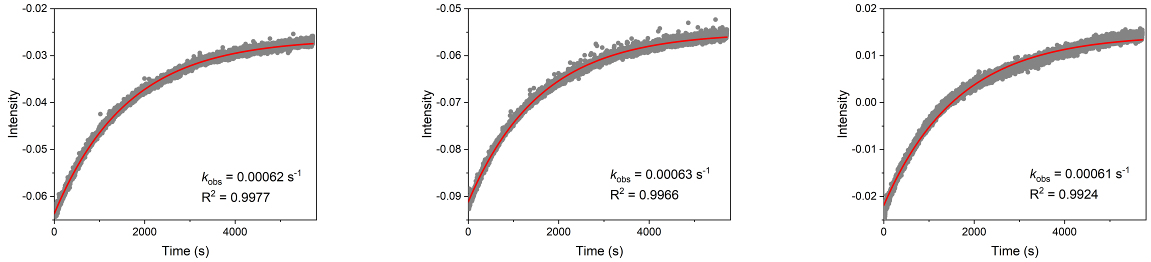

[HEH$_2$] = [Et$_3$N] = 6 mM

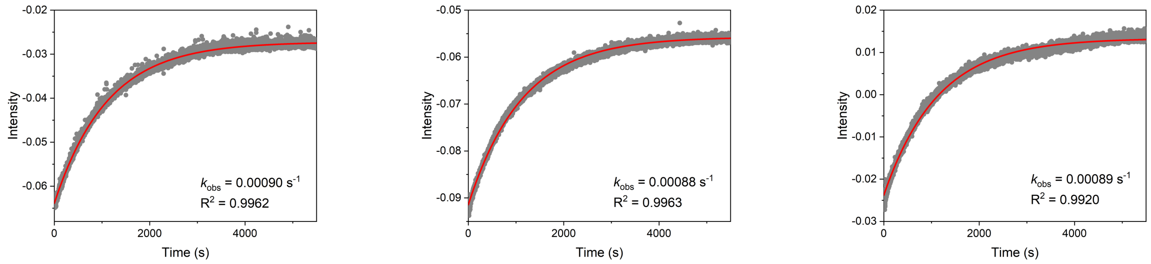

Figure S21. Plots of absorbance at 465 nm over time for the oxidative MS-PCET reaction of dehydrogenation of Hantzsch ester (HEH$_2$) between 0.25 mM **VW$_{12}$** and varied concentrations of [HEH$_2$]/[Et$_3$N] in MeCN at 20 °C with (gray) raw data and (red) fitting curve, along with fit-derived k_{obs}.

Temperature = 0 °C

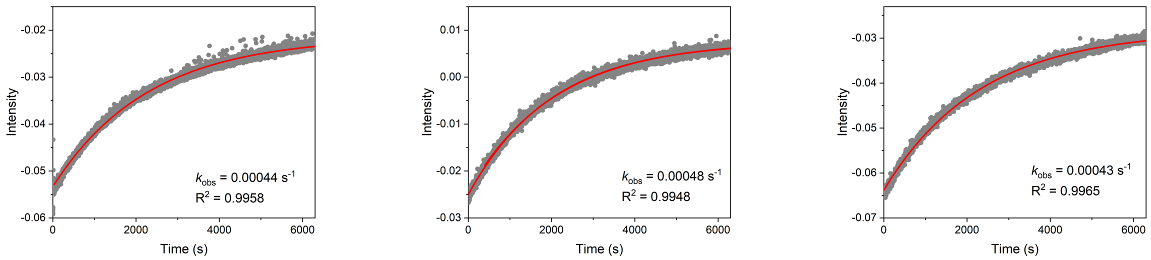

Temperature = 10 °C

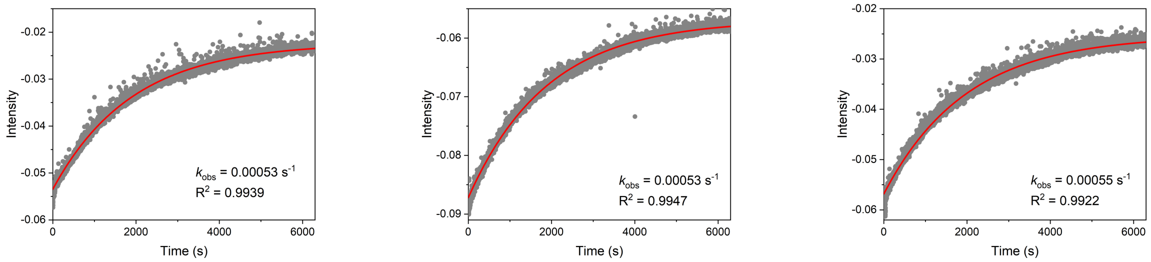

Temperature = 20 °C

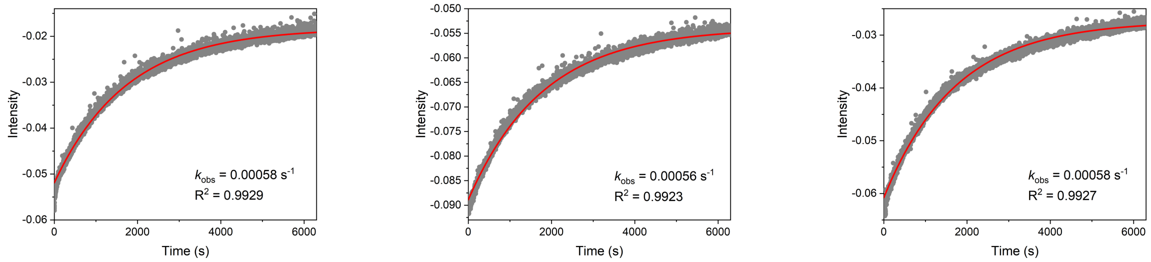

Temperature = 30 °C

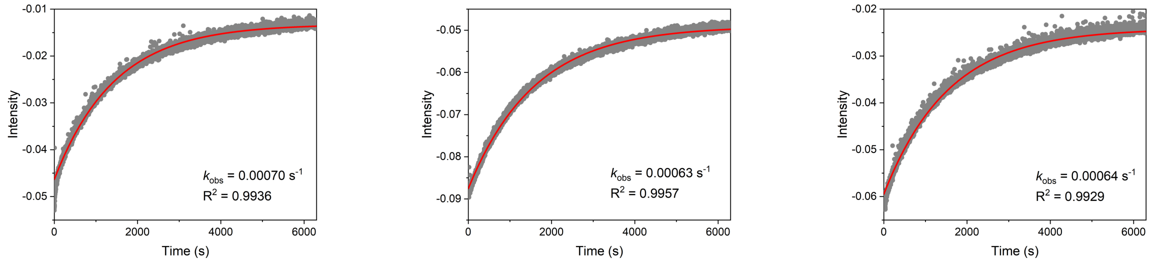

Figure S22. Plots of absorbance at 465 nm over time for the oxidative MS-PCET reaction of dehydrogenation of Hantzsch ester (HEH_2) between 0.25 mM $\mathbf{VW_{12}}$ and 3.75 mM [HEH_2]/[Et_3N] in MeCN at varied temperature between 0 and 30 °C with (gray) raw data and (red) fitting curve, along with fit-derived k_{obs}.

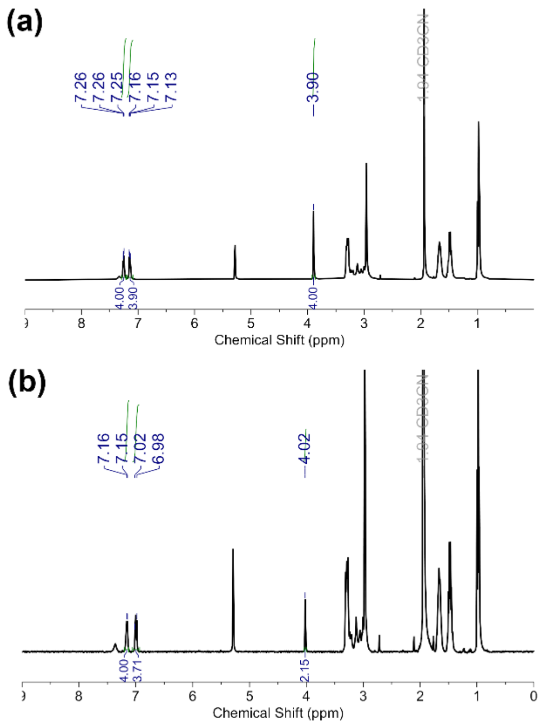

Figure S23. ^{1}H NMR of the reaction mixture of $\mathbf{VW_{12}}$/TMG reagent pair with (a) 9,10-dihydroanthracene and (b) xanthene in MeCN-d_3, while showing no reactivity.

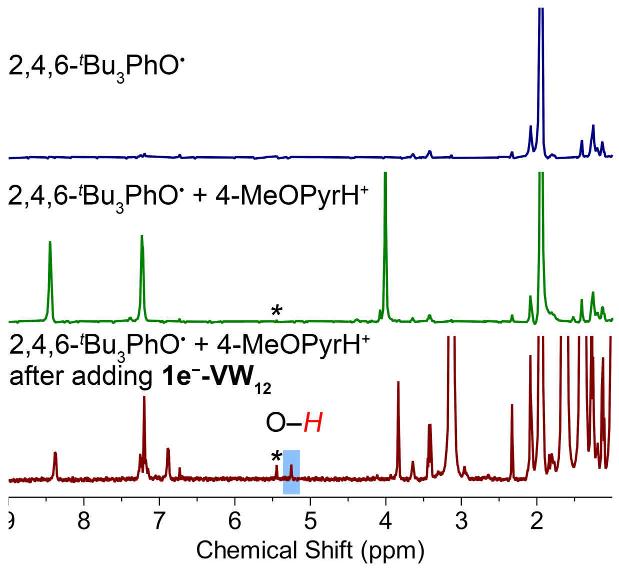

Figure S24. ^{1}H NMR of the reaction mixture of (blue) 2,4,6-^{t}Bu$_3$PhO$^•$ radical, (green) 2,4,6-^{t}Bu$_3$PhO$^•$ radical and 4-MeOPyrH$^+$, and (red) 2,4,6-^{t}Bu$_3$PhO$^•$ radical, 4-MeOPyrH$^+$, and **1e$^-$-VW$_{12}$** in MeCN-*d*$_3$, showing the formation of TTBP with O–H signal highlighted in blue. The asterisk indicates the trace impurity of dichloromethane.

[2,4,6-^{t}Bu$_3$PhO$^•$] = [4-MeOPyH$^+$] = 3 mM

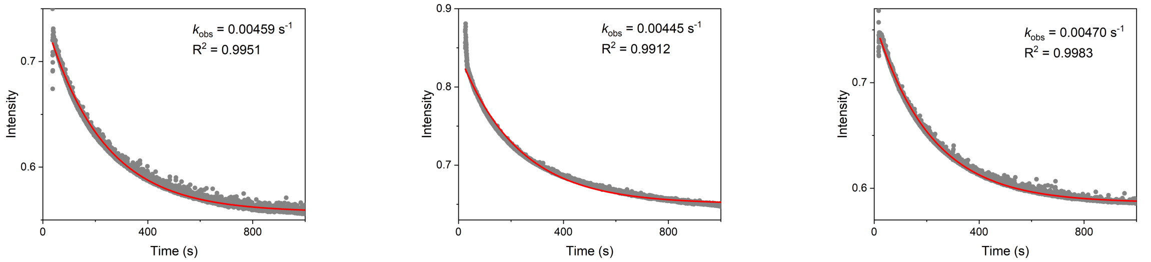

[2,4,6-^{t}Bu$_3$PhO$^•$] = [4-MeOPyH$^+$] = 3.75 mM

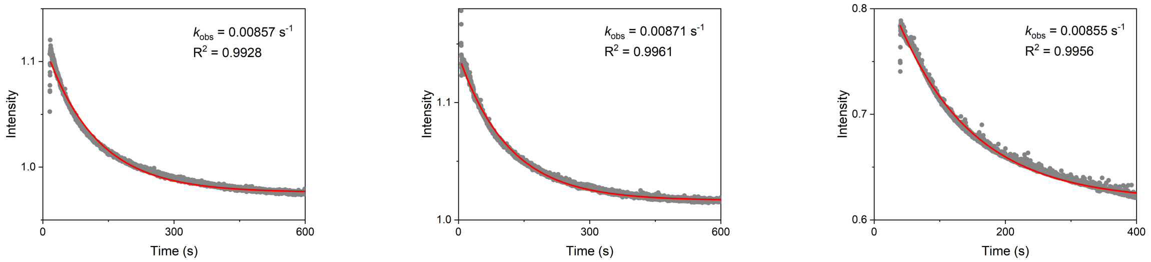

[2,4,6-^{t}Bu$_3$PhO$^•$] = [4-MeOPyH$^+$] = 4.5 mM

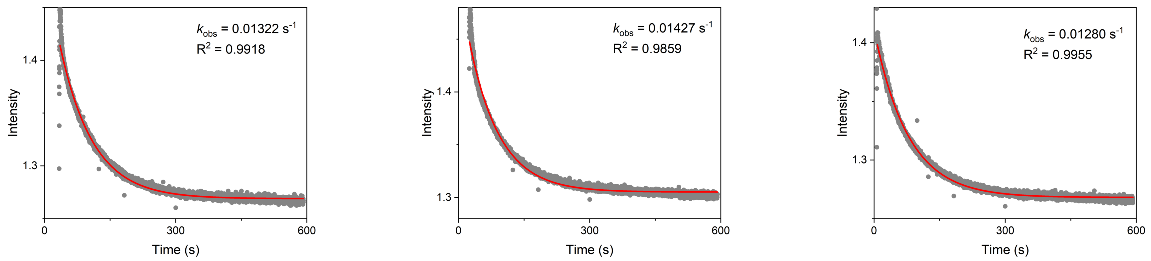

[2,4,6-^{t}Bu$_3$PhO$^•$] = [4-MeOPyH$^+$] = 6 mM

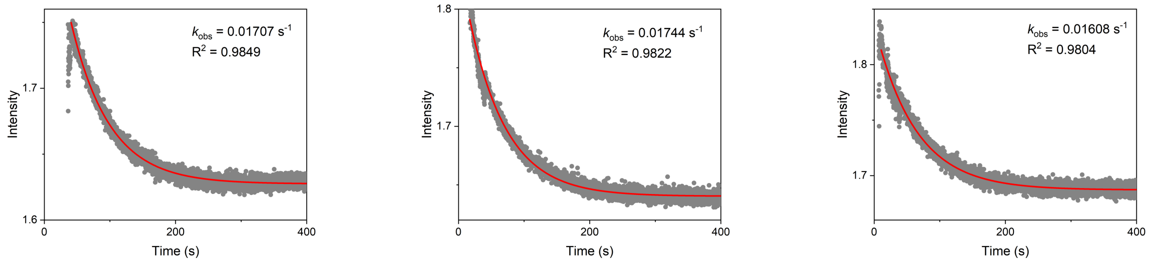

Figure S25. Plots of absorbance at 626 nm over time for the reductive MS-PCET reaction of hydrogenation of 2,4,6-^{t}Bu$_3$PhO$^•$ between 0.25 mM **VW$_{12}$** and varied concentrations of [2,4,6-^{t}Bu$_3$PhO$^•$]/[4-MeOPyrH$^+$(BF$_4^-$)] in MeCN at 20 °C with (gray) raw data and (red) fitting curve, along with fit-derived k_{obs}.

[2,4,6-$^{t}Bu_3PhO^{\bullet}$] = [PyH^{+}] = 3 mM

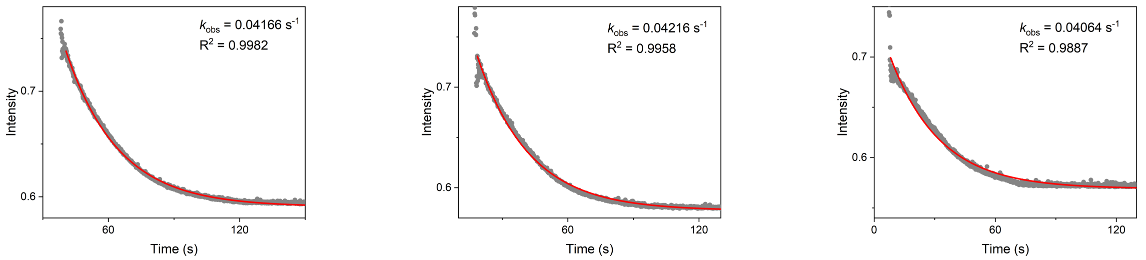

[2,4,6-$^{t}Bu_3PhO^{\bullet}$] = [PyH^{+}] = 3.75 mM

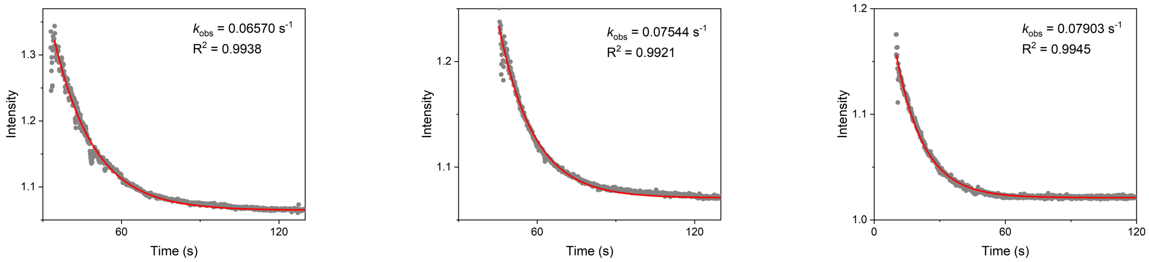

[2,4,6-$^{t}Bu_3PhO^{\bullet}$] = [PyH^{+}] = 4.5 mM

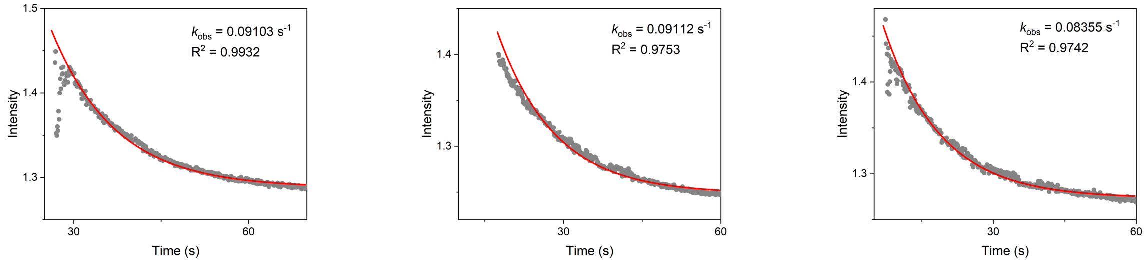

Figure S26. Plots of absorbance at 626 nm over time for the reductive MS-PCET reaction of hydrogenation of 2,4,6-$^{t}Bu_3PhO^{\bullet}$ between 0.25 mM $\mathbf{VW_{12}}$ and varied concentrations of [2,4,6-$^{t}Bu_3PhO^{\bullet}$]/[$PyrH^{+}(BF_4^{-})$] in MeCN at 20 °C with (gray) raw data and (red) fitting curve, along with fit-derived k_{obs}.

[2,4,6-^{t}Bu$_3$PhO$^•$] = [2-PicH$^+$] = 3 mM

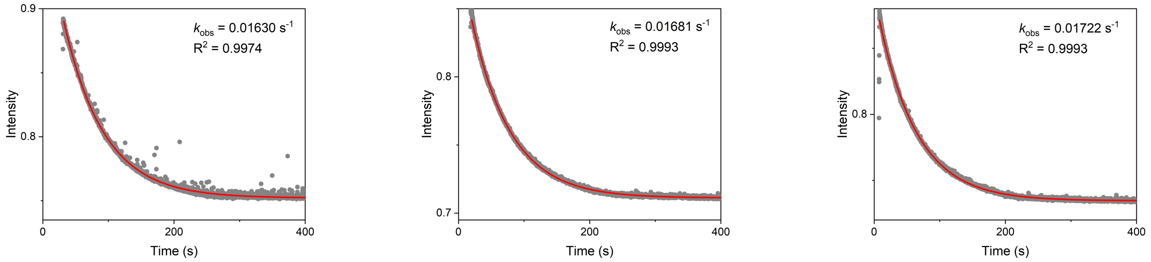

[2,4,6-^{t}Bu$_3$PhO$^•$] = [2-PicH$^+$] = 3.75 mM

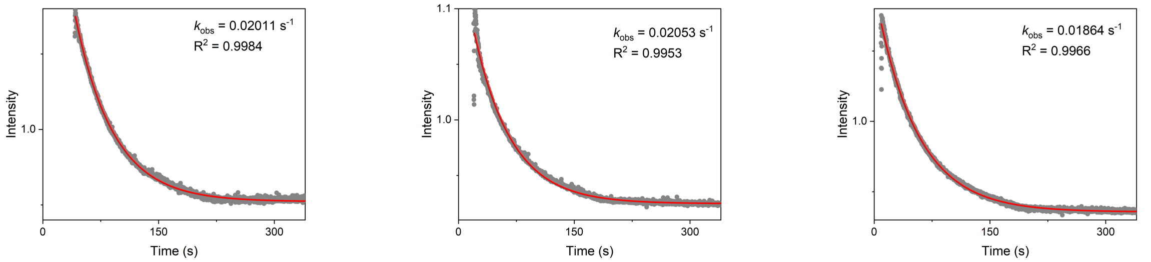

[2,4,6-^{t}Bu$_3$PhO$^•$] = [2-PicH$^+$] = 4.5 mM

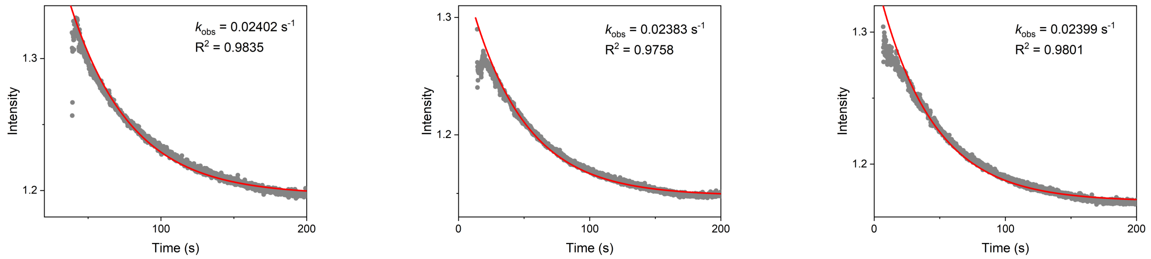

Figure S27. Plots of absorbance at 626 nm over time for the reductive MS-PCET reaction of hydrogenation of 2,4,6-^{t}Bu$_3$PhO$^•$ between 0.25 mM **VW$_{12}$** and varied concentrations of [2,4,6-^{t}Bu$_3$PhO$^•$]/[2-PicH$^+$(BF_4^-)] in MeCN at 20 °C with (gray) raw data and (red) fitting curve, along with fit-derived k_{obs}.

[2,4,6-^{t}Bu$_3$PhO$^•$] = [BimH$_2^+$] = 3 mM

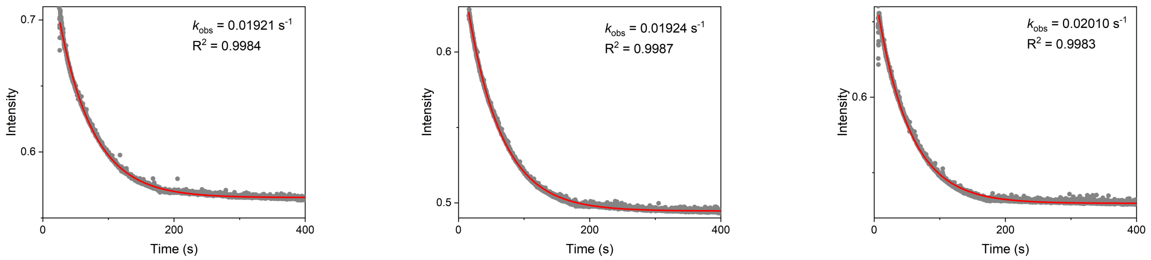

[2,4,6-^{t}Bu$_3$PhO$^•$] = [BimH$_2^+$] = 3.75 mM

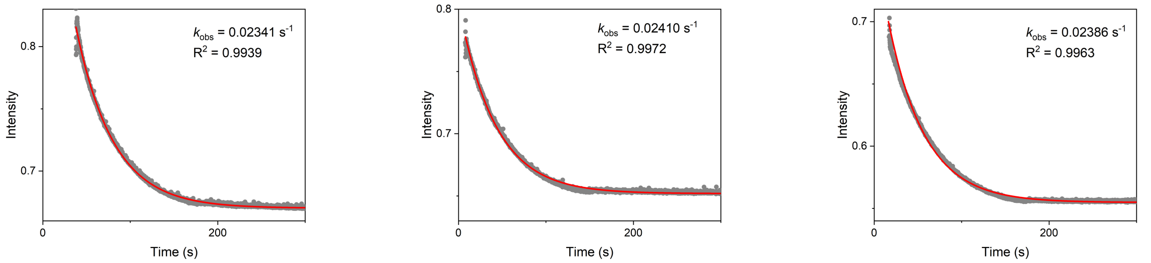

[2,4,6-^{t}Bu$_3$PhO$^•$] = [BimH$_2^+$] = 4.5 mM

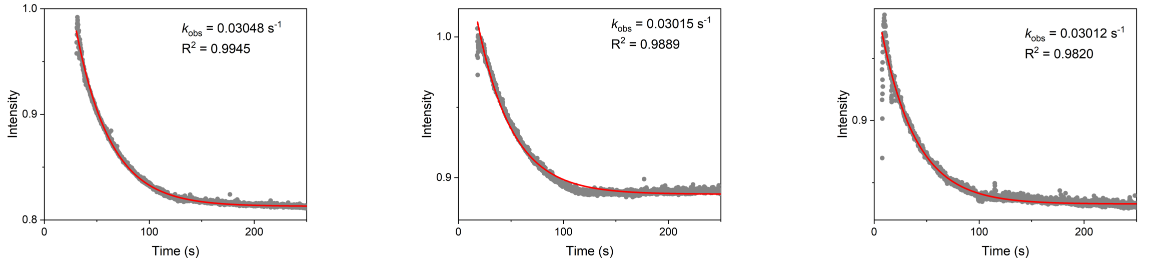

Figure S28. Plots of absorbance at 626 nm over time for the reductive MS-PCET reaction of hydrogenation of 2,4,6-^{t}Bu$_3$PhO$^•$ between 0.25 mM **VW$_{12}$** and varied concentrations of [2,4,6-^{t}Bu$_3$PhO$^•$]/[BimH$_2^+$(BF$_4^-$)] in MeCN at 20 °C with (gray) raw data and (red) fitting curve, along with fit-derived k_{obs}.

[2,4,6-^{t}Bu$_3$PhO$^•$] = [ImH_2^+] = 3 mM

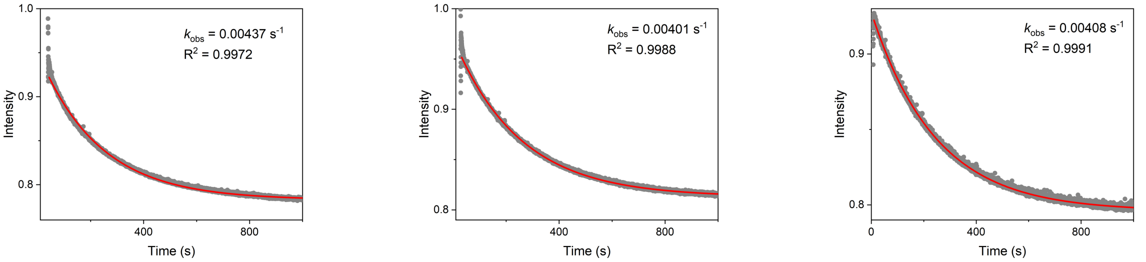

[2,4,6-^{t}Bu$_3$PhO$^•$] = [ImH_2^+] = 3.75 mM

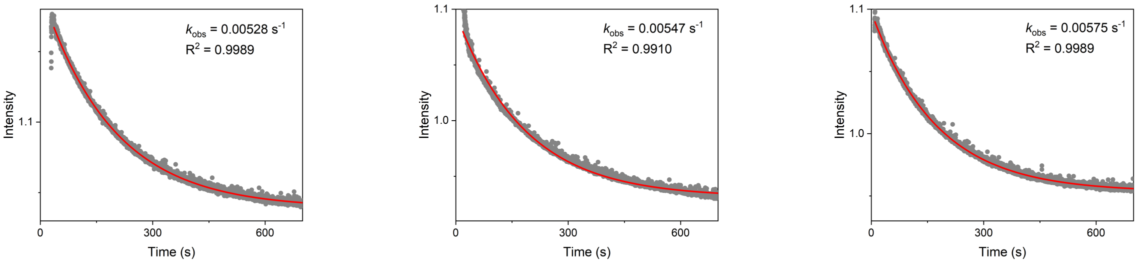

[2,4,6-^{t}Bu$_3$PhO$^•$] = [ImH_2^+] = 4.5 mM

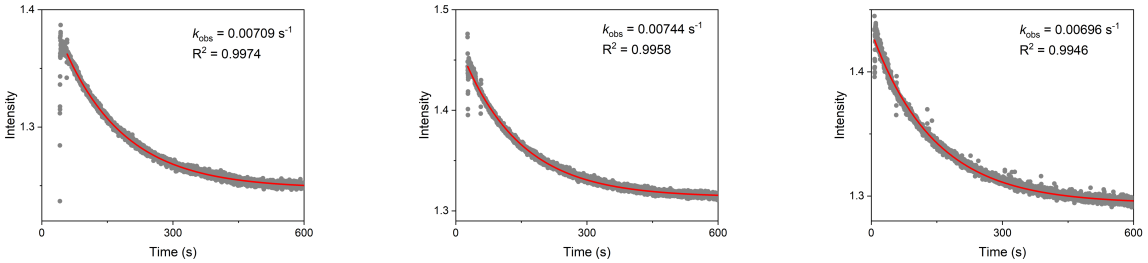

Figure S29. Plots of absorbance at 626 nm over time for the reductive MS-PCET reaction of hydrogenation of 2,4,6-^{t}Bu$_3$PhO$^•$ between 0.25 mM **VW$_{12}$** and varied concentrations of [2,4,6-^{t}Bu$_3$PhO$^•$]/[ImH_2^+(BF_4^-)] in MeCN at 20 °C with (gray) raw data and (red) fitting curve, along with fit-derived k_{obs}.

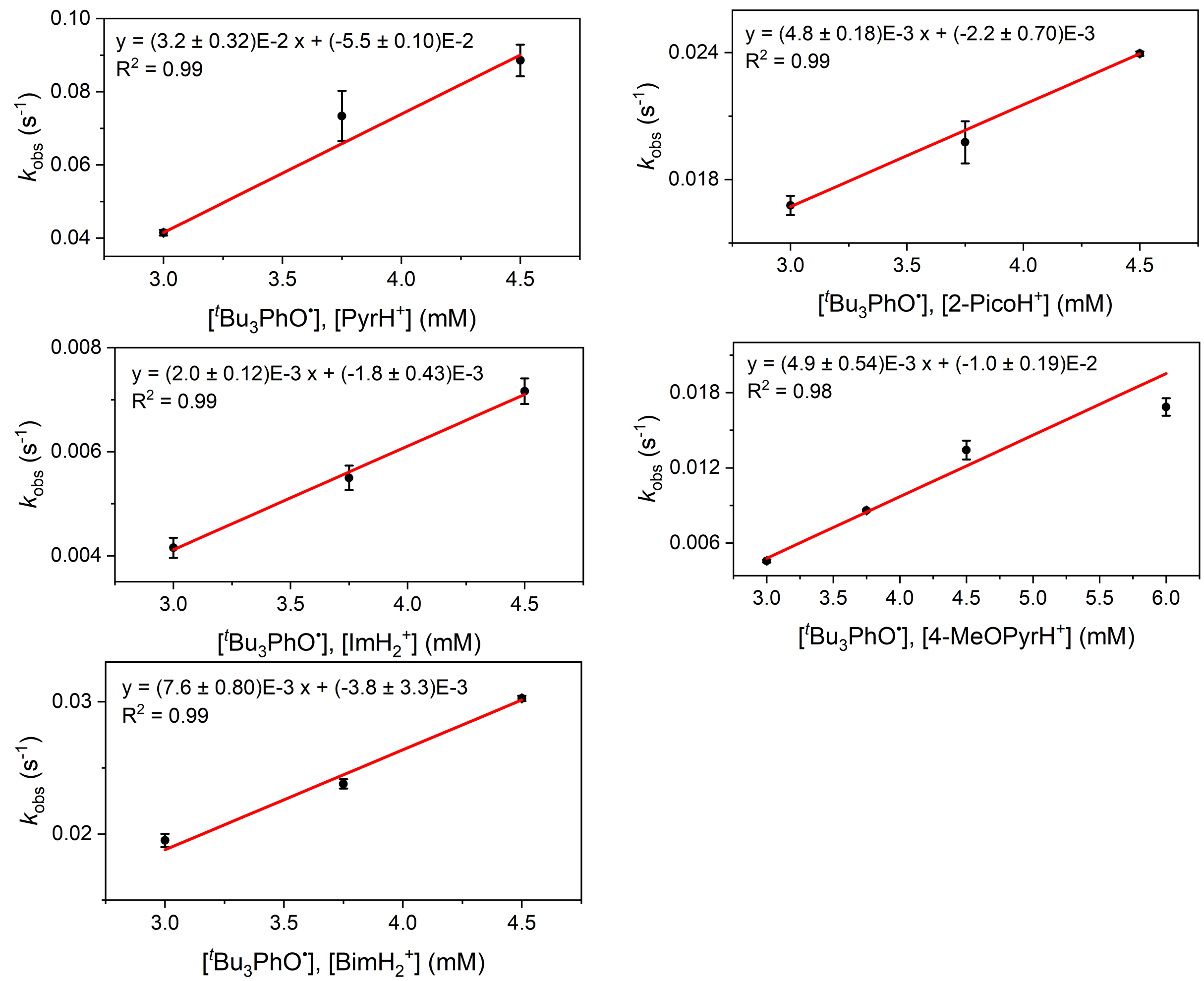

Figure S30. Plots of k_{obs} versus the concentrations of 2,4,6-^{t}Bu$_3$PhO$^•$ and different acids.

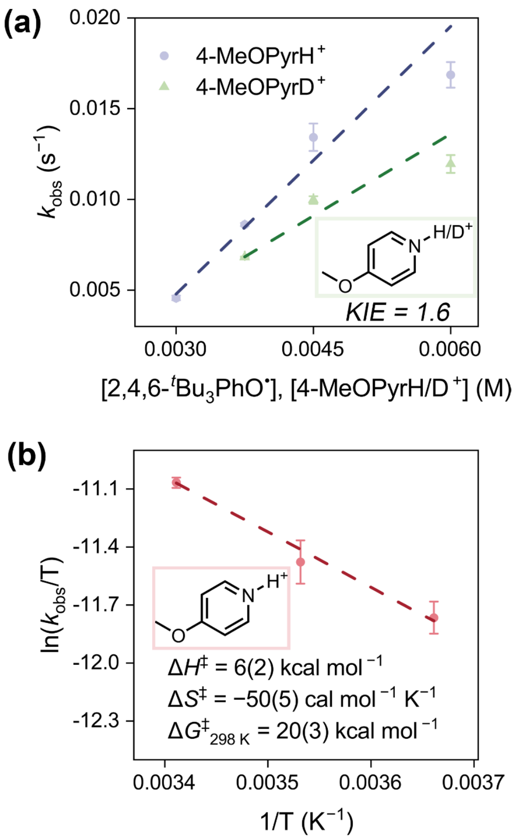

Figure S31. (a) Plots of k_{obs} with respect to the concentrations of [2,4,6-^{t}Bu$_3$PhO$^•$] with [4-MeOPyrH$^+$(BF_4^-)] or [4-MeOPyrD$^+$(OTf$^-$)] (OTf = trifluoromethanesulfonate) at 20 °C, showing the KIE value of 1.6. (b) Eyring plots of the MS-PCET hydrogenation reaction of 2,4,6-^{t}Bu$_3$PhO$^•$ in MeCN by 0.25 mM **1e$^-$-VW$_{12}$**/3 mM 4-MeOPyrH$^+$ between 0 and 20 °C.

[2,4,6-^{t}Bu$_3$PhO$^•$] = [4-MeOPyD$^+$] = 3.75 mM

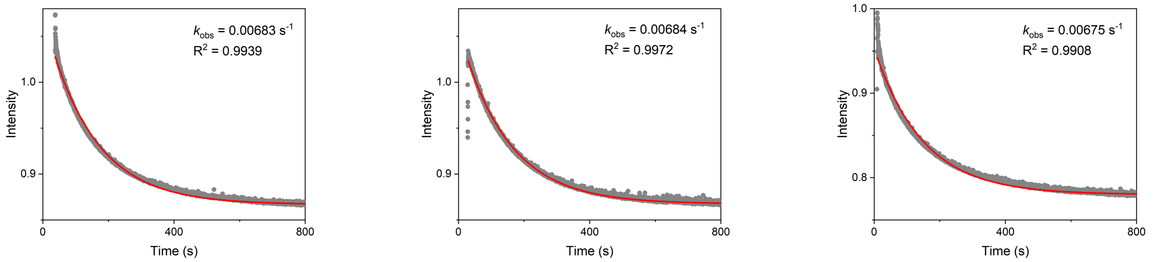

[2,4,6-^{t}Bu$_3$PhO$^•$] = [4-MeOPyD$^+$] = 4.5 mM

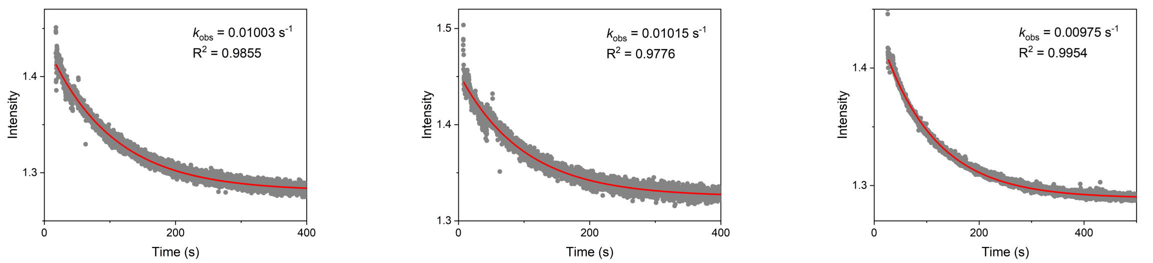

[2,4,6-^{t}Bu$_3$PhO$^•$] = [4-MeOPyD$^+$] = 6 mM

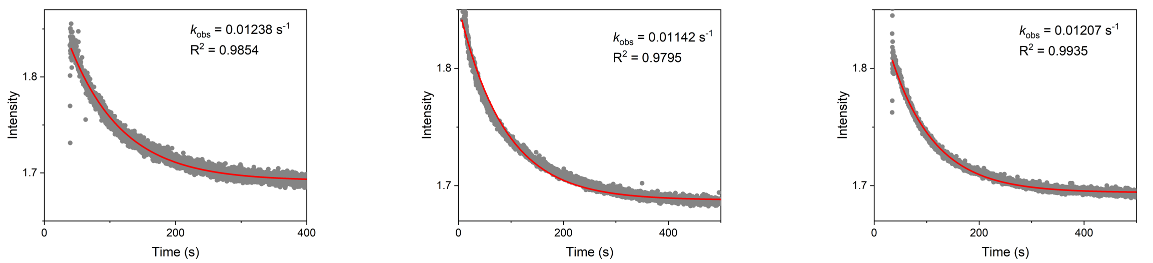

Figure S32. Plots of absorbance at 626 nm over time for the reductive MS-PCET reaction of hydrogenation of 2,4,6-^{t}Bu$_3$PhO$^•$ between 0.25 mM **VW$_{12}$** and varied concentrations of [2,4,6-^{t}Bu$_3$PhO$^•$]/[4-MeOPyrD$^+$(OTf$^-$)] in MeCN at 20 °C with (gray) raw data and (red) fitting curve, along with fit-derived k_{obs}.

Temperature = 0 °C

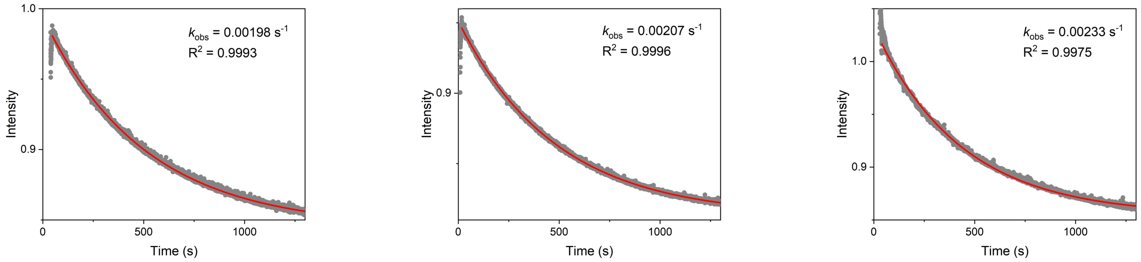

Temperature = 10 °C

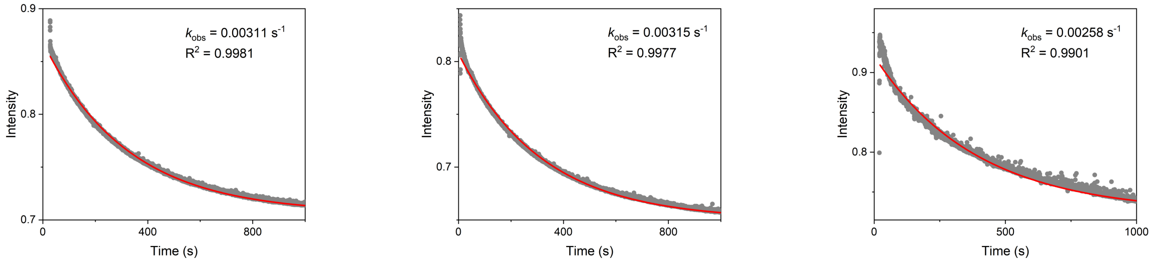

Figure S33. Plots of absorbance at 626 nm over time for the reductive MS-PCET reaction of hydrogenation of 2,4,6-^{t}Bu$_3$PhO$^•$ between 0.25 mM **VW$_{12}$** and 3 mM [2,4,6-^{t}Bu$_3$PhO$^•$]/[4-MeOPyrH$^+$(BF$_4^−$)] in MeCN at varied temperature between 0 and 10 °C with (gray) raw data and (red) fitting curve, along with fit-derived k_{obs}.

REFERENCES

(1) Himeno, S.; Takamoto, M.; Higuchi, A.; Maekawa, M. Preparation and Voltammetric Characterization of Keggin-Type Tungstovanadate $[VW_{12}O_{40}]^{3-}$ and $[V(VW_{11})O_{40}]^{4-}$ Complexes. *Inorg. Chim. Acta* **2003**, *348*, 57–62.
(2) Lu, Z.; Dagar, M.; McKone, J. R.; Matson, E. M. Location of Dopant Dictates Proton–Coupled Electron Transfer Mechanism in Vanadium-Substituted Polyoxotungstates. *Chem. Sci.* **2025**, *16*, 6736–6743.
(3) Manner, V. W.; Markle, T. F.; Freudenthal, J. H.; Roth, J. P.; Mayer, J. M. The First Crystal Structure of a Monomeric Phenoxyl Radical: 2,4,6-Tri-Tert-Butylphenoxyl Radical. *Chem. Commun.* **2008**, *0*, 256–258.
(4) Goldsmith, C. R.; Jonas, R. T.; Stack, T. D. P. C−H Bond Activation by a Ferric Methoxide Complex: Modeling the Rate-Determining Step in the Mechanism of Lipoxygenase. *J. Am. Chem. Soc.* **2002**, *124*, 83–96.
(5) Saeed, S.; Lu, Z.; Matson, E. M.; Augustyn, V. Expanding Proton-Insertion Coupled Electron Transfer into Tungsten Oxides to Non-Aqueous Organic Acid Electrolytes. *Chem. Commun.* **2025**, *61*, 18356–18359.
(6) Tshepelevitsh, S.; Kütt, A.; Lõkov, M.; Kaljurand, I.; Saame, J.; Heering, A.; Plieger, P. G.; Vianello, R.; Leito, I. On the Basicity of Organic Bases in Different Media. *Eur. J. Org. Chem.* **2019**, *2019*, 6735–6748.
